# Deep short-read sequences facilitated identification of seven putative drought tolerance genes in a genome-wide association study in soybean

**DOI:** 10.3389/fpls.2025.1661547

**Published:** 2025-12-16

**Authors:** Atit Parajuli, Ramesh Chethri, Iman Saha, Micheline N. Ngaki, Cecelia Ryden, Madeline Thompson, Qingfeng Xing, Liang Dong, Madan K. Bhattacharyya

**Affiliations:** 1 Department of Agronomy, Iowa State University, Ames, IA, United States; 2 Microelectronics Research Center, Iowa State University, Ames, IA, United States; 3 Department of Electrical and Computer Engineering, Ames, IA, United States

**Keywords:** soybean, drought tolerance, GWAS, leaf-flipping, transpiration, wearable plant sensors

## Abstract

Drought stress significantly limits soybean yield, especially if it occurs during flowering and early pod development stages. To better understand the genetic mechanisms of drought tolerance in legume soybean, we conducted genome-wide association studies (GWAS) for (i) leaf-flipping and (ii) transpiration traits. A short list of seven candidate drought tolerance genes was generated from 67 GWAS-discovered genes by determining if (i) mutations alter structure and function of candidate genes, (ii) the genes are drought responsive due to mutations in putative *cis*-acting elements, and (iii) they were shown to contribute towards drought tolerance. We used rainout shelters to ensure drought stress and wearable plant sensors to measure leaf-surface humidity and temperature to determine transpiration rates. From GWAS of 240 soybean accessions for the leaf-flipping trait, we identified three candidate drought tolerance genes: (i) a *thaumatin-like protein* gene, the tea homologue of which regulates the root hair development and drought tolerance in *Arabidopsis*, (ii) a chloroplast *isopropyl malate synthase* gene that plays an important role in root development for drought tolerance; (iii) transcriptionally regulated *glycinol 2-dimethyltransferase* gene. Investigation of 47 accessions for transpiration rates revealed two candidate transcriptionally regulated drought-responsive genes encoding α-tubulin and phosphoenolpyruvate carboxykinase (PCK). The α-tubulin was shown to control stomatal opening, while PCK improves water retention by closing stomata during drought stress. An uncharacterized DUF1118 containing protein and HAT5 homeodomain-leucine zipper protein could also regulate transpiration during drought stress. In this study, we have demonstrated that short-read sequences and transcriptomic data facilitate identification of strong candidate drought tolerance genes.

## Introduction

Soybean (*Glycine max* (L) Merr.) is a key source of U.S. agricultural economy generating a cash receipt of $59 billion in 2023 (USDA-ERS, 2023). It has been the major source of protein and oil, and is widely used as animal feed as well as for producing industrial products including lubricants and biodiesel (Liu et al., 2020). However, soybean production has been suppressed substantially by drought. Based on a comprehensive 50-year study, it was concluded that 12.8% of the annual soybean yield variation is due to drought stress (Zipper et al., 2016). The rising global temperature driven by climate change has rapidly depleted soil moisture leading to more frequent drought episodes (Malhi et al., 2021). Prolonged drought has severe repercussions on crop yield (Leng and Hall, 2019) posing a significant threat to global food security (Seleiman et al., 2021). Although there is a modest increase in global arable land (Gupta et al., 2020), the demand for irrigation water has increased substantially (Miller, 2002). Development of drought-tolerant cultivars represents a promising strategy to sustain crop production in drought-prone regions.

Drought tolerance is a complex trait. Tolerant genotypes limit the maximum transpiration rates during drought stress and may develop deeper root systems to access the moisture from a lower water table. Additionally, these genotypes maintain high chlorophyll content, delay proline accumulation, reduce leaf size and gas exchange, and sustain high relative water content during drought stress (Hossain et al., 2014; Prince et al., 2015). In soybean, drought effects can be visible both in seedling and reproductive stages (Han et al., 2022; Igiehon et al., 2021). Short and infrequent episodes of drought do not affect productivity and quality in soybean (Vaghar et al., 2020); however, prolonged and recurrent episodes, especially during reproductive stages, are detrimental to the soybean crop (Zhang et al., 2021).

Physiological symptoms of drought in soybean include wilting and leaf-drooping that appear under water stress due to decreased cell water potential and cell turgidity (Wang et al., 2022). Increased water stress can reduce soybean seed germination rate, damage photosynthetic apparatus, reduce plant height, pod number and yield (Kausar et al., 2012). Selection of genotypes that can efficiently utilize the available water during drought stress is the first step towards improving soybean for drought tolerance (Sinclair, 2018; Ye et al., 2018). For example, slow canopy wilting resulting from reduced transpiration rates has been considered as an important trait for breeding drought-tolerant soybean cultivars (Menke et al., 2024).

Canopy wilting in soybean is the direct response of plants to decreased turgor pressure. Breeders exploit this common trait to identify drought tolerant genotypes (Menke et al., 2024). It has been shown that under a high vapor pressure deficit (VPD), tolerant soybean genotypes reached a maximum transpiration rate of 2.0 kPa, while sensitive genotypes showed increased transpiration rates greater than 2.0 kPa (Fletcher et al., 2007). Decreased transpiration during higher VPD allows moisture conservation and improved water use efficiency (Fletcher et al., 2007). The genetics of slow canopy wilting is highly complex and governed by over 30 quantitative trait loci (QTL) (Abdel-Haleem et al., 2011, 2012; Hwang et al., 2015; Ren et al., 2020; Ye et al., 2019), identified through study of segregating materials generated from biparental crosses and over 80 single nucleotide polymorphism (SNP) loci (Aleem et al., 2024; Kaler et al., 2017; Li et al., 2023) discovered through genome-wide association studies (GWAS) of natural variants. Unfortunately, none of these genetic loci for drought tolerance has been molecularly characterized.

Drought stress suppresses the accumulation of miR166, which is conserved across the land plants (Yadav et al., 2021). In rice, miR166 knockdown mutants exhibit the rolled-leaf phenotype, reduced stomatal conductance and reduced transpirations rates under drought stress (Zhang et al., 2018). miR166a regulates the lateral root number and drought tolerance in maize (Zhang M, et al., 2022). It has been shown that the knockdown of miR166 sustains higher pod set and seed yield under water-stress conditions in soybean (Zhao C, et al., 2024).

A suite of plant wearable sensors capable of continuously monitoring crop physiology and micro-climatic parameters is currently available (Yan et al., 2024). These sensors, typically attached to plant parts such as the lower leaf surface (Li M, et al., 2022), have recently been deployed to track changes in relative humidity (RH), biopotential, nutrient level (Church et al., 2017; Diacci et al., 2020; Nagamine et al., 2023), transpiration rates, and even reactive oxygen species under various stress conditions (Lan et al., 2020; Oren et al., 2017; Singh et al., 2025; Yin et al., 2021). The sensor attached to the leaf surfaces records the transpiration-induced RH and temperature fluctuations. It offers advantages over conventional RH sensors, including easy installation, low cost, and lightweight design. The functional layer of the sensor consists of a patterned graphene-based composite created using a roll-to-roll patterning method and a metal thin-film resistive element whose resistance changes with water vapor. The sensor is structured as a strip transferred onto gas/vapor permeable tape, which ensures adhesion to the leaf surface while enabling efficient gas/vapor exchange for photosynthesis. The sensor is flexible and does not interfere with plant growth. Field deployments have demonstrated its ability to detect differences in transpiration between fertilized and unfertilized maize plants (Ibrahim et al., 2022).

Soybean exhibits leaf flipping and leaf clamping phenotypes under water stress conditions. The flipped leaves during drought stress expose the silvery-green ventral side to reflect the sunlight to reduce the leaf temperature. This can significantly reduce the photosynthesis process and water use. Similarly, leaves are folded during drought stress, known as leaf clamping, to conserve water. The leaf clamping minimizes the surface area exposed to the sun resulting in reduced photosynthesis activity, leaf temperature and water use (Casteel, 2012). The drought-tolerant genotypes exhibit tighter stomatal control, allowing slow and more regulated water release to maintain optimum leaf temperature and metabolic activity (Ye et al., 2019). The soybean genotypes, such as ADT 1 and CAR 1260 with slower transpiration rates exhibit enhanced yield stability under combined drought and heat stress (Parasuraman et al., 2023).

In this study, we conducted genome-wide association studies (GWAS) for two drought stress-related traits: (1) leaf-flipping, a less studied visible drought-avoidance phenotype, and (2) transpiration rates using a wearable plant sensor. Integrating transpiration rates gathered by wearable plant sensors every 30 minutes with leaf-flipping phenotyping enables simultaneous, high-resolution assessment of physiological and morphological drought response under the rainout shelters (Yin et al., 2021; Yin and Dong, 2024);. While the leaf-flipping trait provides a clear visual drought response of drought sensitive lines, wearable sensors delivers continuous, quantification of plant water loss along with leaf-surface temperature. Investigation of just 47 accessions for transpiration rates using this plant sensor revealed two candidate transcriptionally regulated drought-responsive genes encoding α-tubulin and phosphoenolpyruvate carboxykinase (PCK). The α-tubulin was shown to control stomatal opening, while PCK improves water retention by closing stomata during drought stress (Sheoran et al., 2014; Zhang et al., 2017). Thus, our novel approach of using plant wearable sensors led to identification of two putative drought responsive genes that could regulate stomatal opening during drought stress.

## Results

### Leaf-flipping phenotypes of a diverse collection of soybean accessions

A collection of 240 diverse soybean lines, including 172 Plant Introduction (PI) lines that were reported to be highly diverse based on their genetic variation (Valliyodan et al., 2021) and 68 improved soybean germplasm lines/cultivars developed at the Iowa State University and a few commercial cultivars, were included in this study. Some of the selected PI lines have been previously identified as drought tolerant lines. During the severe drought of 2023 (**Supplementary Table 1**), we observed leaf flipping phenotype among some of the soybean accessions under rainfed conditions in addition to under the artificially created drought conditions in rainout shelters.

**Figure 1:**
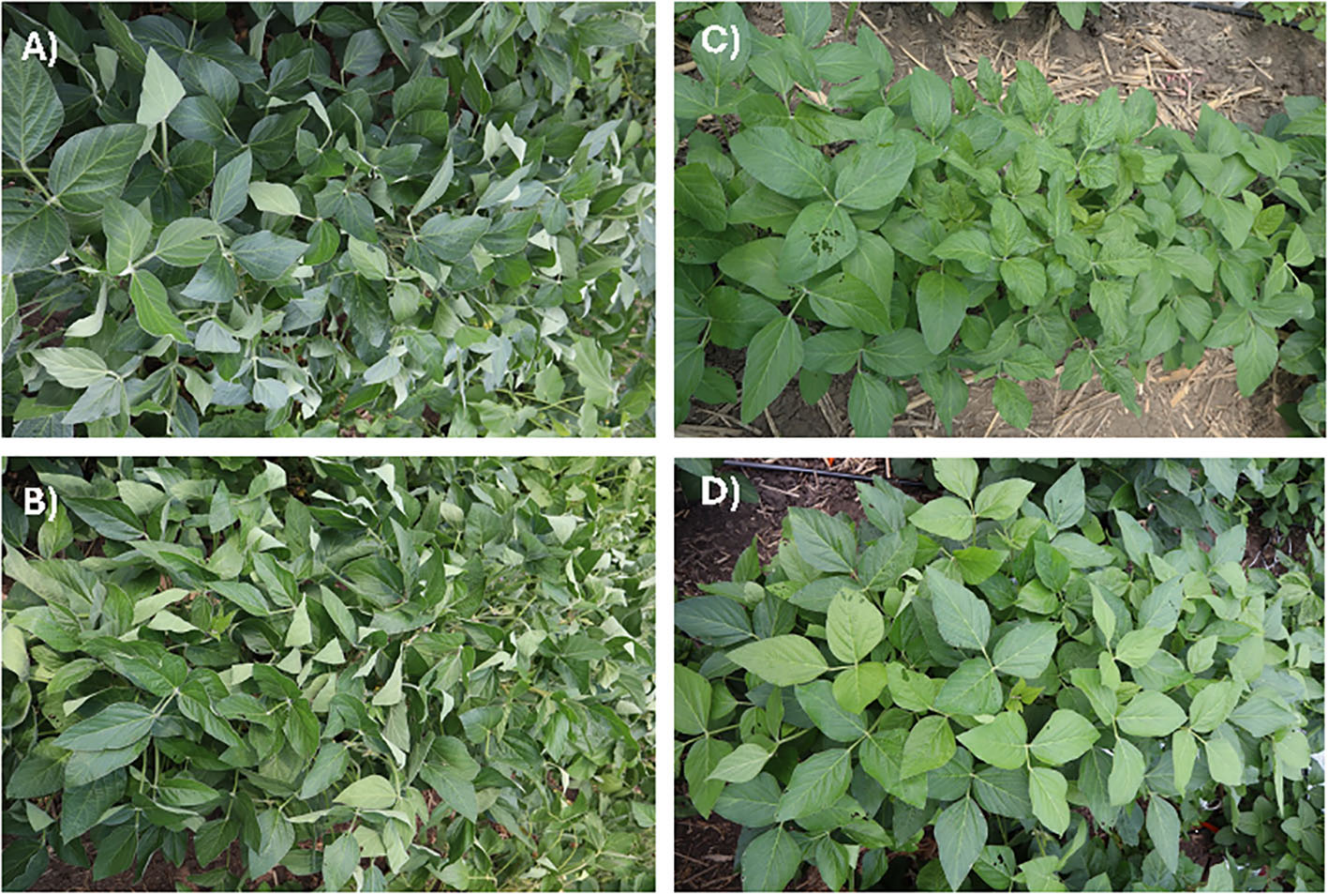
Leaf flipping phenotypes observed under induced drought in rainout shelters in two drought-sensitive accessions, **(A)** AR18SCN, and **(B)** IAR1000SDS/SCN. **(C)** PI 391583 and **(D)** S146205DRTRes are two drought tolerant accessions with a not so obvious leaf-flipping phenotype.

The leaf flipping phenotype of soybean (**Figure 1**) was more pronounced under the rainout shelters and the scores ranged from 0.5 to 54 with a mean of 12.4 (**Supplementary Figure 1**; **Supplementary Table 2**) as compared to that under rainfed conditions where the scores ranged from 1.5 to 14.5 with a mean of 6.1 (**Supplementary Figure 1**; **Supplementary Table 3**). Severe drought conditions under rainout shelters presumably enhanced the leaf flipping scores.

### Genotype data

Genotyping of the 240 lines was carried out using Khufu sequencing technology at Hudson Alpha Institute for Biotechnology for approximately one genome equivalent. The Williams 82 Version 4 reference genome sequence was used for calling the single nucleotide polymorphisms (SNPs) among the lines. After imputation of some of the missing data, 71,560 SNPs were identified. However, after filtering for minor allele frequency (<5%) and heterozygosity (>5%), 30,843 SNPs were available for GWAS of the leaf-flipping trait among the 240 accessions. The SNPs were uniformly distributed among most chromosomes with a minimum of 20 SNPs in each mega base pair DNA. However, for some chromosomes like 1, 5, 8, 12, 14 and 20, uneven distribution of SNPs was observed (**Supplementary Figure 2**).

### Population structure of the genotypes used for scoring the leaf-flipping trait

**Figure 2:**
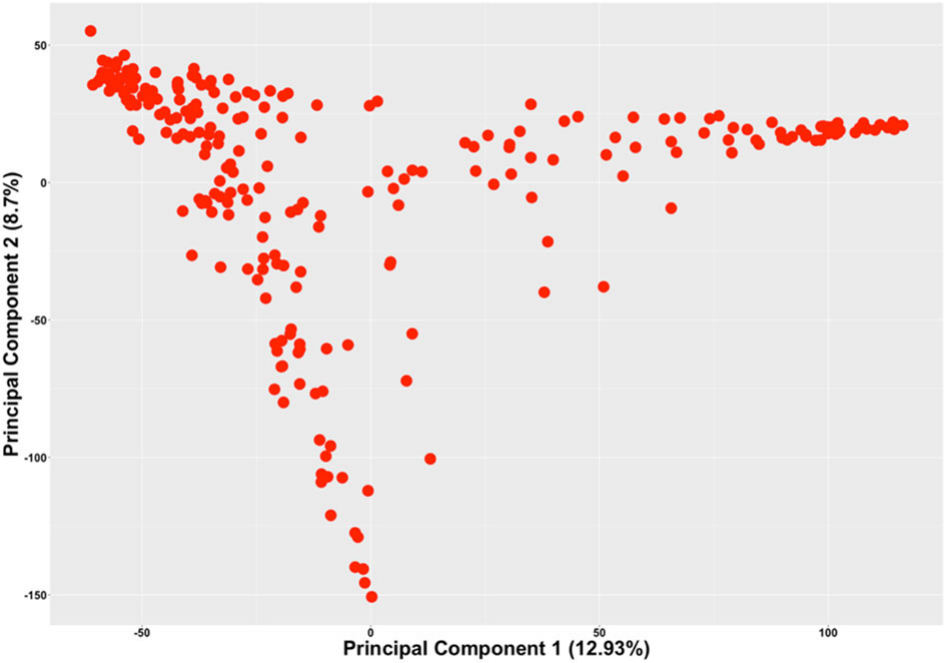
The scatterplot of the 240 genotypes based on the first two principal components generated from the PCA of the 240 genotypes using 30,843 SNPs.

The current study included highly diverse plant introduction (PI) lines, developed cultivars and germplasm lines at Iowa State University, and elite commercial cultivars. As such, population structure confounds the association study, if not accounted for in the association model. Principal component analysis (PCA) was conducted to study the population structure among the 240 genotypes using 30,843 SNPs. The first two principal components (PCs), explaining 21.63% of the genotypic variation, were used to depict the population structure of the 240 accessions (**Figure 2**). Three distinct clusters among the 240 lines indicate the presence of a distinct population structure among the 240 accessions and were accounted for in the association model.

### Genome-wide association study of the leaf-flipping trait

GWAS was conducted for the leaf flipping trait scores collected for each of the 240 genotypes. Two types of scores were generated for each genotype: (i) total scores of all plants of a genotype in a plot; or (ii) scores/plant of a genotype. The data were collected for plants grown under (i) rainout shelters or (ii) rainfed conditions with no rainout shelters. We used 30,843 SNPs for GWAS. We incorporated the first three principal components as covariates in the model to account for population stratification and minimizing confounding effects in our GWAS. Bayesian-Information and Linkage-disequilibrium Iteratively Nested keyways (BLINK) model was used in Genome Association and Prediction Integrated Tool 3 (GAPIT3) for the final association study of the trait. The *p*-value threshold was set to the modified Bonferroni corrected *p*-value.

**Figure 3:**
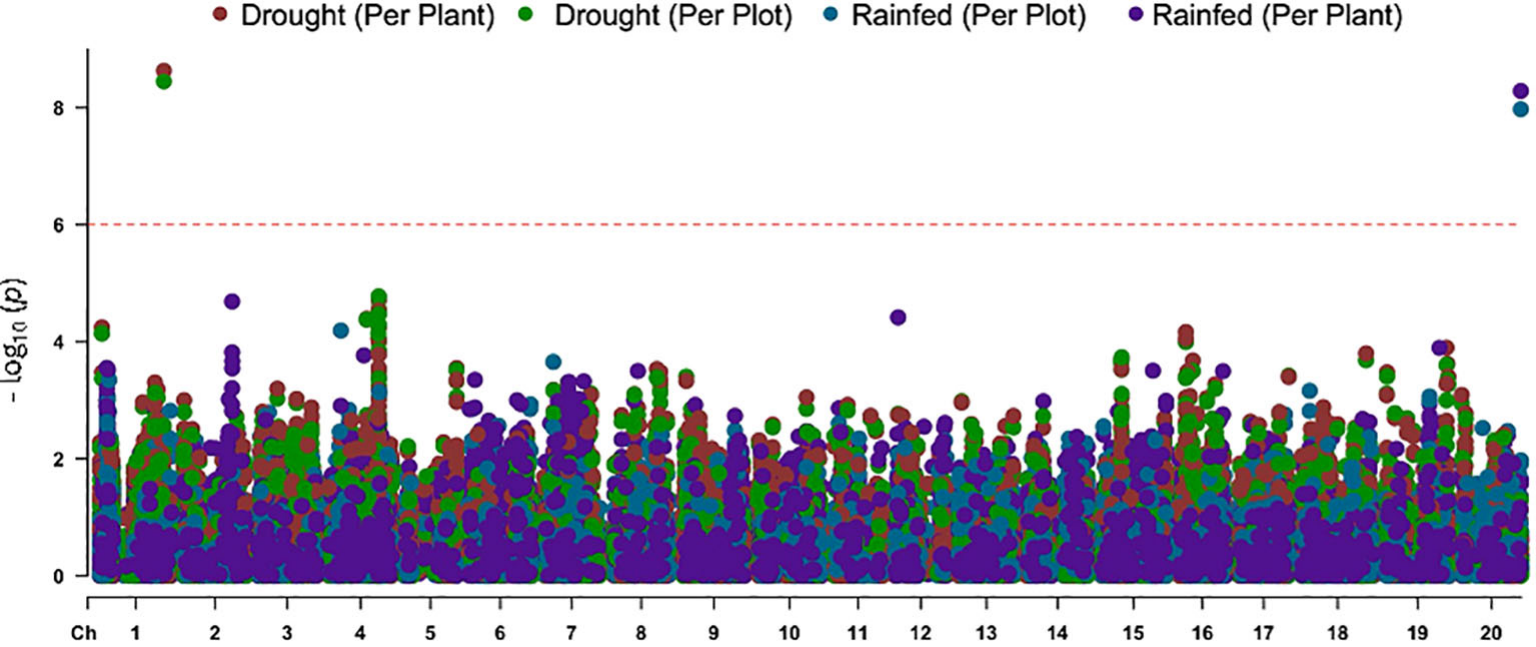
The Manhattan plot showing the associations of two SNP loci with the leaf-flipping phenotype mapped to Chromosomes 1 and 20. The horizontal broken line represents the Bonferroni corrected *p*-value threshold. Drought (Per Plant), phenotypic scores of single plants under rainout shelters; Drought (Per Plot), phenotypic scores based on images of individual plots under rainout shelters; Rainfed (Per Plant), phenotypic scores of individual plants under rain-fed conditions; and Rainfed (Per Plot), phenotypic scores based on images of individual plots of the rainfed crop.

GWAS revealed a SNP on Chromosome 1 that was significantly associated with the variation in the leaf flipping trait among the 240 accessions grown under rainout shelters. The *p*-value of the association was slightly lower when the leaf flipping scores per plant was used as opposed to the scores per plot (**Figure 3**). The variation in leaf-flipping scores for the plants grown under rainfed conditions with no rainout shelters was significantly associated with two SNPs, separated by 2,041 bp, on Chromosome 20 (**Figure 3**). The SNP mapped to Chromosome 1 explained about 18.12% of the phenotypic variation while two SNPs on Chromosome 20 explained about 12.14% and 10.15%, respectively. All the three identified SNPs are located within genes, one SNP in each of *Glyma.01G165800, GmHk_20G059303* and *Glyma20g245300* genes.

**Figure 4:**
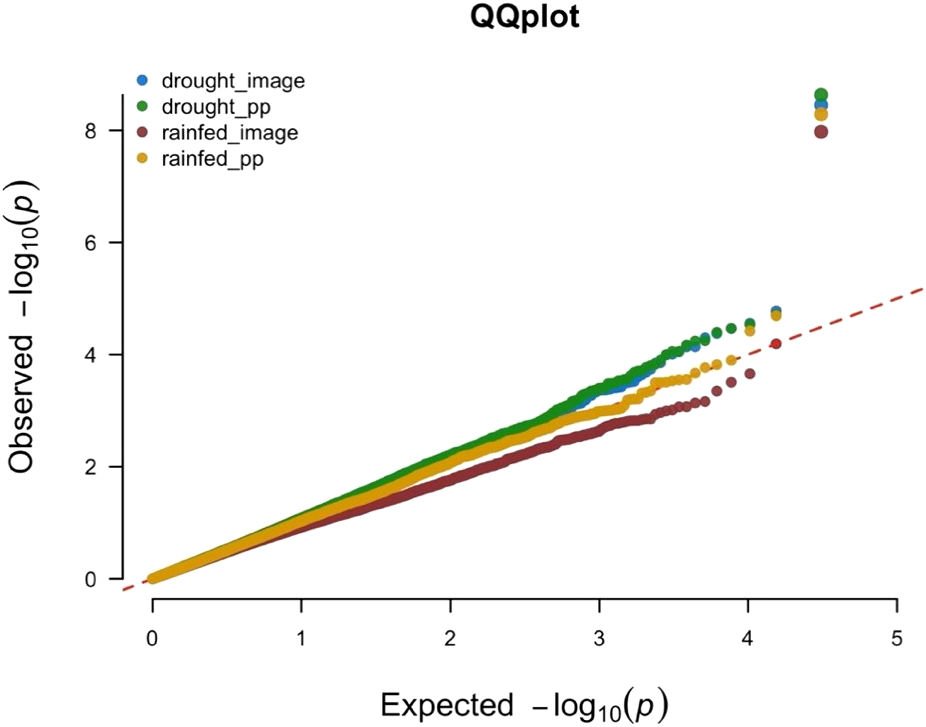
Quantile-Quantile plot for the Manhattan plot presented in **Figure 3**. The four different phenotypic data sets are represented by four different colors. Blue color for the scores collected/plot from plants grown under rainout shelters (drought_image), green color for the scores collected/plant from plants grown under rainout shelters (drought_pp), while brown color is used for data collected/plot from plants grown under no rainout shelters (rainfed_image) and gold color is used for data collected/plant from plants grown under no rainout shelters (rainfed_pp).

The QQ-plot indicated effective control of false positive result due to population stratification as the *p*-value distribution showed strong alignment with the expectation under null, particularly across majority of the distribution (**Figure 4**). The minor inflation observed at the tail of the distribution is consistent with the presence of the signals. This successful control is attributed to the inclusion of the first three principal components as fixed effects covariates in the GWAS model.

### Annotation of the putative candidate genes associated with the leaf-flipping trait

Putative candidate gene annotation was performed using the gene coordinate files from the Williams 82 reference genome assembly (version 4). The significant SNPs associated with the leaf-flipping phenotype mapped to coding regions of two genes on Chromosomes 1 and 20. The SNP on Chromosome 1 is localized to the coding sequence of the *Glyma.01G165800* gene, named *GmTP1*, encoding a thaumatin-like protein. Two physically linked SNPs on Chromosome 20 localized to the coding sequences of an unannotated gene (*GmHk_20G059303*) and *Glyma20g245300*, named *GmIMS1*, encoding an isopropylmalate synthase, respectively. Notably, *Glyma.20G245300* has three transcript variants. The genes encoding a thaumatin-like protein and an isopropylmalate synthase were found to be associated with drought tolerance in earlier studies (Muoki et al., 2021; Schaufelberger et al., 2019).

### Characterization of the SNPs associated with the leaf-flipping trait

The SNP identified within the *GmTP1* gene resulted in a non-synonymous mutation leading to substitution of cysteine (C) with phenylalanine (F). While the reference Williams 82 genome carried the nucleotide for the cysteine residue, the alternate allele with phenylalanine instead of cysteine was more frequent in the population suggesting that the Williams 82 could carry the favorable allele for drought tolerance (**Supplementary Figure 3**). The average phenotypic image score for the genotypes with unfavorable allele was 10.5, while that of the favorable allele (reference allele) was 7.1, indicating a possible role of cysteine in the structure-function of the favorable allele encoding a thaumatin-like protein for enhancing drought tolerance in soybean (**Supplementary Figure 3**).

The significant SNP mapped to Chromosome 20 caused a nonsynonymous mutation at the *GmIMS1* gene resulting in substitution of a cysteine residue with the amino acid arginine. In this case, the leaf flipping scores among the genotypes carrying the allele with the cysteine residue was higher (6.5) than the scores (4.4) of the genotypes with the allele containing the arginine residue. Only 34 of the 240 accessions carry the arginine residue suggesting a possible role of arginine in the structure-function of an isopropylmalate synthase that contributes to drought tolerance in soybean (**Supplementary Figure 4**).

An additional significant SNP, located in the open reading frame of the unannotated gene *GmHk_20G059303*, resulted in proline-to-leucine substitution in the encoded hypothetical protein GmHk_20G059303. Here, the leaf flipping score among the genotypes carrying the allele with the proline residue was higher (6.5) in comparison to that for the leucine score (5.5). The allele frequency for the reference allele was 0.6 while that for the alternate allele was 0.4 (**Supplementary Figure 5**). The hypothetical protein encoded by this gene has shown no significant identity with any known proteins. This protein is unlikely to contribute towards drought tolerance because the association of this gene with the leaf-flipping trait variation is loose.

**Figure 5:**
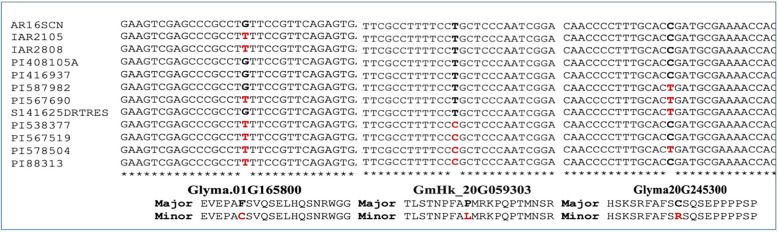
The segregation of the alleles for three SNPs detected in the coding sequences of three genes among 12 soybean accessions.

To validate the alleles of three SNP loci mapped to Chromosomes 1 and 20, we investigated genome sequences of 12 soybean accessions representing the two extreme values of the leaf-flipping phenotypes. We sequenced each genotype to 30X genome equivalent DNA. We observed segregation of the two alleles in each of the three SNP loci (**Figure 5**) among the selected 12 soybean accessions. Among the 12 genotypes, the association of the alleles of *GmHk_20G059303* with those of the *Glyma20g245300* carrying the significant SNP mapped to Chromosome 20 is weak (**Figures 3**, **5**).

We also looked at the haploblock region containing the significant SNPs on Chromosome 1 and Chromosome 20 to investigate if there were any differentially expressed drought-responsive genes (Shin et al., 2015). We identified 11 genes within the haploblock on Chromosome 1 covering about 133 kb region (**Supplementary Table 4**). Among the 11 genes none of them were found to be differentially expressed in a transcriptomic study of drought stress in soybean (Shin et al., 2015). Similarly, the haploblock carrying two significant SNPs on Chromosome 20 spans about 122 kb containing 19 genes. Among the 19 genes, *Glyma.20G245100* named *GmG2MT* encoding the glycinol 2-dimethyltransferase was found to be differentially expressed in response to drought stress or dehydration and should be considered as potential gene contributing to drought tolerance (Shin et al., 2015; Jahan et al., 2019; Li et al., 2022; **Supplementary Tables 5**). Using the deep ~ 30 X genome Illumina short read sequences of 12 selected lines including both drought sensitive and tolerant lines, we identified two indels (AATTAT, TTATATA) at the upstream region of the *GmG2MT* gene. AATTAT and TTATATA motifs mapped to the indels contain domains for binding to transcription factors, zinc finger homeodomain (ZF-HD) and AP2-ERF.

### The transpiration rates among 47 diverse soybean accessions

**Figure 6:**
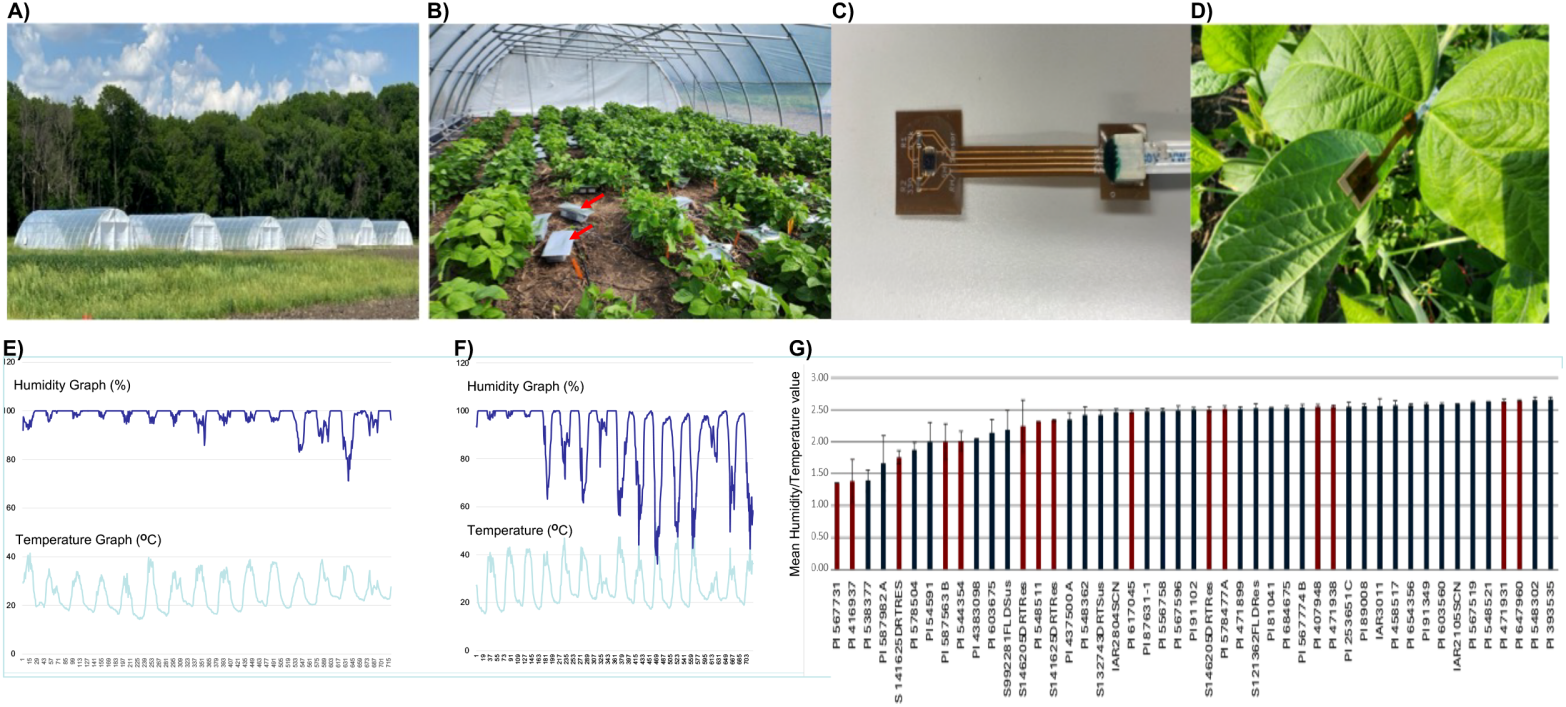
Recording of humidity and temperature on ventral leaf surfaces collected by wearable plant sensors under rainout shelters. **(A)** Six rainout shelters used in this study; **(B)** soybean plants with sensors and data loggers; **(C)** a wearable plant sensor; **(D)** a wearable plant sensor attached to a ventral leaf surface; **(E)** data recorded by a sensor on a leaf of a drought sensitive line; **(F)** data recorded by a sensor on a leaf of a drought tolerant line; and **(G)** humidity/temperature ratio as a drought tolerance index of 47 genotypes. The H/T values of genotypes, previously reported to be drought tolerant, are presented in red bars **(G)**.

A subset of 47 soybean lines, including some known drought tolerant lines were selected from the 240 accessions for studying the transpiration rates using wearable plant sensors in drought conditions induced under the rainout shelter numbers 3 and 4 (from the left in **Figure 6A**) serving as two blocks or replications (**Figures 6B, C**). We had to study a smaller population (n = 47) including some drought tolerant lines due to lack of availability of a large number of wearable plant sensors. The plant wearable sensors were attached to the lower side of the third leaves and were changed to younger leaves (**Figure 6D**). The sensors were connected to the data logger containing SD cards (**Figure 6B**) that stored the temperature and humidity data every 30 minutes. A sensor was attached to one leaf of each of the three individual plants of a genotype in each replication.

The ratio of humidity (H) to temperature (T) during midday was used as an index (H/T) for the rate of transpiration of individual plants. The H/T values ranged from 1.37 to 2.67 (**Supplementary Table 6**). Of the three lines with the low H/T indices, two lines, PI 567731 and PI 416937, were previously reported to be drought tolerant (Ye et al., 2019) (**Figures 6E–G**). Of these, PI 567731 showed a very low leaf-flipping phenotypic score under rainout shelters (**Supplementary Table 2**).

### Population structure of the population used for scoring the transpiration rates

The population structure of the 47 selected lines studied using wearable plant sensors was determined by conducting principal component analysis (PCA). For this, 17,042 SNPs identified after filtering for minor allele frequency (< 5%) and heterozygosity (> 5%) were used. The first two PCs explain 23.57% of the genotypic variation (**Supplementary Figure 6**). The scatter plot developed from the first and second PCs exhibited a clear population structure among the 47 genotypes. We used the first three principal components as covariates in our GWAS model to remove the confounding effect resulting from this population structure in the association study. Bayesian-Information and Linkage-disequilibrium Iteratively Nested keyways (BLINK) model was used in GAPIT3 for the final association study of the trait. The *p*-value threshold was determined using the Bonferroni correction method.

### Genome-wide association study of the transpiration rate

**Figure 7:**
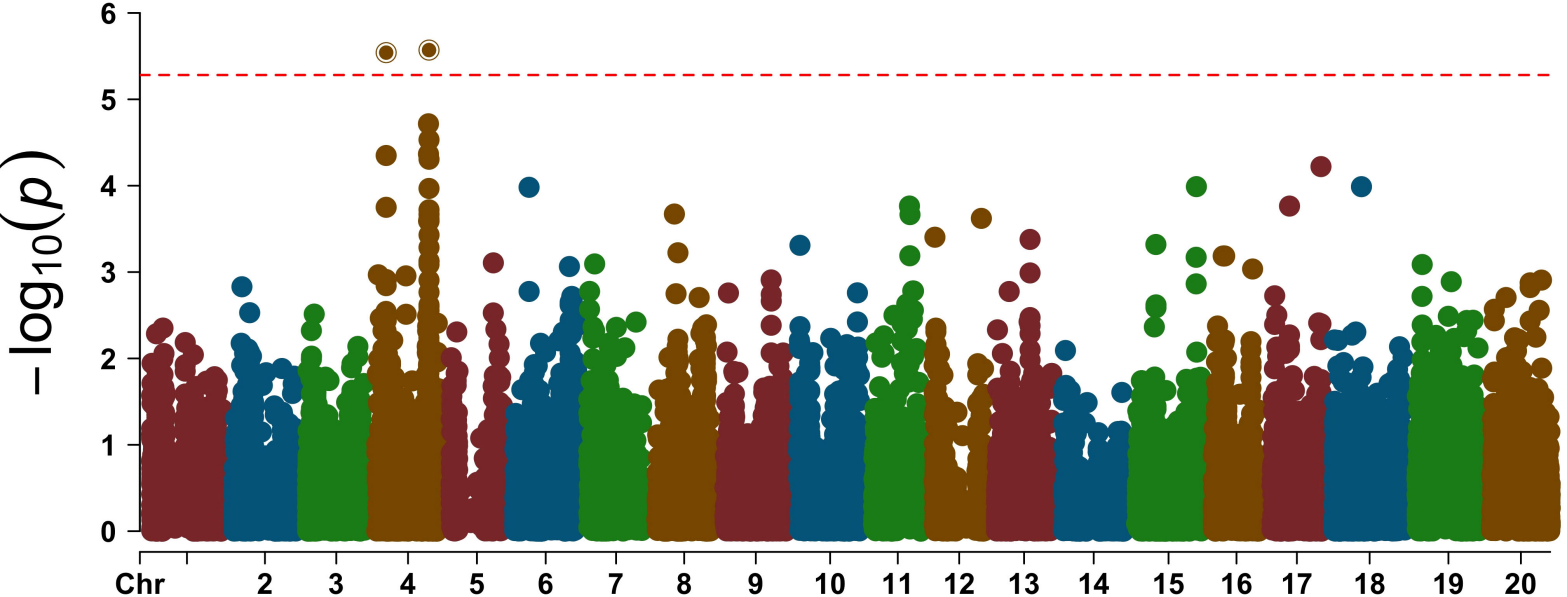
Manhattan plot showing the association of two SNPs with the H/T indices of the 47 accessions. The horizontal dashed red line represents the Bonferroni corrected threshold value, and the two significant SNPs are shown above the threshold line.

**Figure 8:**
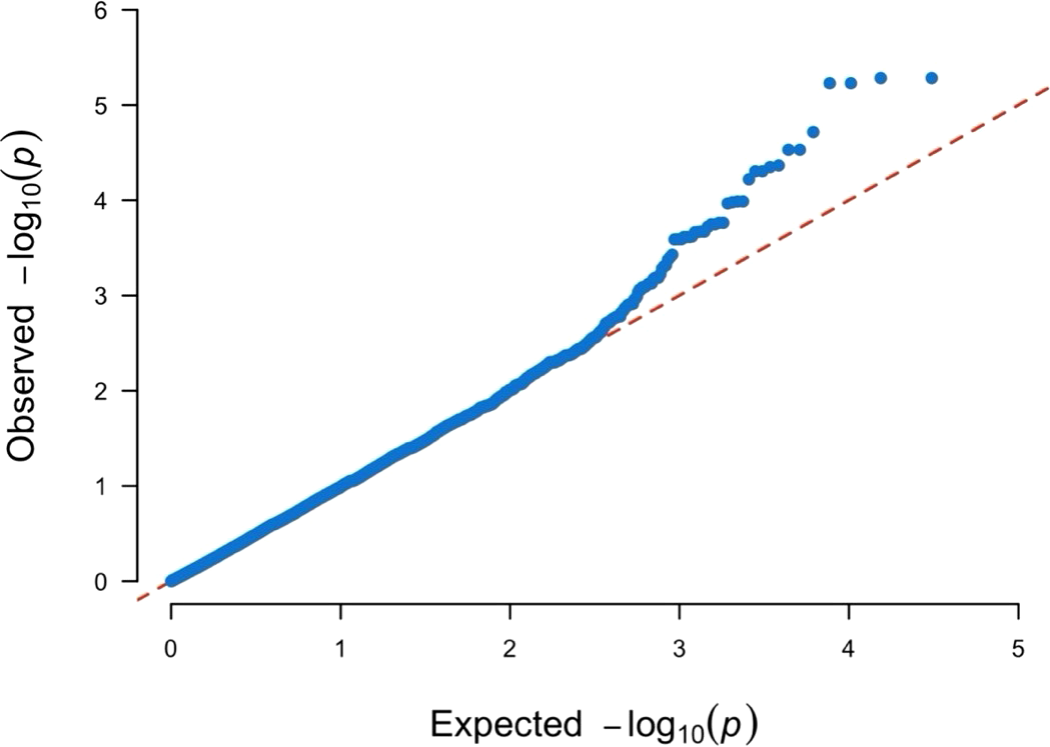
Quantile-Quantile plot for the Manhattan plot presented in **Figure 7**. The observed *p*-values follow the uniform distribution as expected with the significant SNPs (top right corner) deviating away from the expectation.

In GWAS of the H/T indices using 17,042 SNPs, we identified two significant SNPs mapped to Chromosome 4 (**Figures 6**, **7**). The QQ-plot (**Figure 8**) confirmed that population stratification was effectively controlled in the GWAS. The *p*-value distribution was a strong match to the null expectation, indicating a low rate of false positives. The slight upward deviation at the tail end suggests the presence of true signals. This successful adjustment is credited to the use of the first three principal components as covariates in the analysis.

Of the two SNPs mapped to Chromosome 4, one SNP mapped to the northern arm of Chromosome 4, while the other SNP mapped to the southern arm of Chromosome 4. We also look into the phenotypic variation of the H/T ratio over 47 genotypes with the respective significant SNP at the northern arm and found that lower values were correlated with reference allele and higher values with the alternate allele (**Supplementary Figure 7**) We investigated the haploblock of 297,437 bp carrying the SNP in the northern arm and 461,479 bp carrying the SNP of the southern arm of Chromosome 4 for putative candidate drought tolerance genes and identified 37 genes.

Among the 37 genes identified within the two haploblocks on Chromosome 4, 21 were found in the northern haploblock and 16 in the southern haploblock (**Supplementary Tables 7**, **8**). We investigated the differential expression patterns of the 37 genes in response to drought stress using published data (Shin et al., 2015). We identified two genes located on the northern haploblock (**Supplementary Table 7**), *Glyma.04G088500* and *Glyma.04G089700* that are differentially expressed during drought stress (Shin et al., 2015). These two genes are considered as putative candidate drought tolerance genes contributing to the regulation of transpiration rates during the drought stress. Moreover, *Glyma.04G088500* was located 92,805 bp upstream while *Glyma.04G089700* was 122,368 bp downstream of the significant SNP identified at the northern haploblock.

To determine the causal mutations for the differential expression of *Glyma.04G088500* named *GmATB1* encoding an α-tubulin 1 protein and *Glyma.04G089700* termed *GmPCK1* encoding a phosphoenolpyruvate carboxykinase (PCK) in response to drought stress, we investigated the up- and down-stream sequences of each gene for possible insertion-deletions (indels) that could carry transcription factor binding sites. The genotypes investigated for H/T indices and sequenced for >20X genome equivalent short-read sequences (**Figure 5**) were considered for identifying the possible indels. We detected indels in the 3’-end sequence or downstream silencer region of *GmATB1* and *GmPCK1*, respectively. In the *GmATB1* gene, the alternate allele had a deletion of 27 base pair CCGGTATGAACTTTATTAATTTAATAA sequence in the 3’-end region of the unfavorable allele (**Supplementary Figure 8**; **Supplementary Table 9**). Using PlantPAN 4.0 program, we were able to show that this sequence carries binding sites for DoF, ZF-HD and ARID transcriptions factors (**Supplementary Table 10**).

For the *GmPCK1* gene, we identified the repetitive sequence ‘TGGGCT’ four times among the six drought tolerant lines and nine times among the two drought sensitive lines (**Supplementary Figure 9**; **Supplementary Table 11**). The repetitive element is predicted to contain several binding sites for transcription factors including NF-YA, NF-YB and NF-YC, and the TCP domain transcription factor binding motif SITEIIATCYTC (**Supplementary Table 12**; Nakaminami et al., 2009). The loss of the transcription factor binding sites could result in the differential expression of the genes during drought stress conditions. In the putative silencer region of *GmPCK1*, the over representation of the TGGGCT sequence could result in the negative regulation of the *GmPCK1 gene by* NF-YA, NF-YB, NF-YC and SITEIIATCYTC transcription factors.

We investigated the haploblock containing a significant SNP (**Figure 7**) on the southern arm of Chromosome 4 for possible functional changes in the 16 genes using at least 20X genome equivalent sequences of seven genotypes which were investigated using the wearable plant sensor. We selected two genes, *Glyma.04G174400* (uncharacterized protein) and *Glyma.04G174467* (*homeobox-leucine zipper protein* HAT5-like) carrying missense mutation and conservative in-frame insertion, respectively, for further study because the alleles of these two genes are associated with the H/T indices among the seven genotypes (**Supplementary Table 8**). These genes were located at 219,607 bp and 216,145 bp upstream of the significant signal identified in the southern haploblock respectively. The mutation in *Glyma.04G174400* was responsible for substation of a proline (P) residue with the positively charged arginine (R) amino acid (**Supplementary Figure 10**). It showed to carry DUF1118 (Domain of unknown function) (**Supplementary Figure 11**). Investigation of this protein in SignalP (https://services.healthtech.dtu.dk/services/SignalP-6.0/) suggested strongly that it’s a secretory protein (*p* < 0.002). We named this putative candidate drought tolerance gene as *pGmDT1 (putative Glycine max drought tolerance 1).* For *Glyma.04G174467* encoding a homeobox-leucine zipper HAT5-like protein, the in-frame insertion of three nucleotides (A → ACTT) resulted in addition of a serine residue to the N-terminal serine-rich motif that is most likely involved in protein structure-function (**Supplementary Figure 12**). We termed this gene *pGmDT2 (putative Glycine max drought tolerance 2).* Both *pGmDT1* and *pGmDT2* are candidate drought tolerance genes and require validation through overexpression and gene knockout studies to establish their possible drought tolerance function.

## Discussion

Drought stress has been a major limiting factor that significantly reduces the potential soybean yield. A prolonged exposure of soybean to drought impedes normal physiology of the crop leading to reduced growth and development. During flowering and reproductive stages, prolonged drought causes significant yield reduction in soybean (Seleiman et al., 2021; Zhang et al., 2021). Drought tolerance is a complex trait governed by a large number of genetic loci (Abdel-Haleem et al., 2011, 2012; Hwang et al., 2015; Ren et al., 2020; Ye et al., 2019; Aleem et al., 2024; Kaler et al., 2017; Li et al., 2023). Apart from uncovering the role of miR166 in drought tolerance, not much is known regarding the molecular basis of the trait in soybean (Zhao et al., 2024).

In this study, we conducted GWAS for two drought stress-related traits: (i) leaf-flipping phenotype and (ii) transpiration rate. A wearable plant sensor was used to determine the humidity and temperature on the ventral or abaxial surface of trifoliate leaves for calculating the humidity/temperature (H/T) index, as measure of transcription rates of 47 soybean accessions.

Rainout shelters were applied to ensure drought stress. The 240 highly diverse soybean accessions were studied using a digital camera for the leaf-flipping trait. The year 2023 had very little rainfall and we were able to observe leaf-flipping phenotypes even without the rainout shelters (**Supplementary Table 1**). The GWAS of the leaf-flipping trait scores revealed two genetic loci: one on Chromosome 1 for the scores collected from the plants grown under the severe drought stressed under rainout shelters; and the other one on Chromosome 20 for the scores collected from the plants that were grown outside the rainout shelters. The phenotypic variations of the leaf-flipping trait were distinct between the soybean plants grown inside and outside of the rainout shelters, with wider range (53.5) and larger mean (9.6) for the population grown under severe drought generated in the rainout shelters and with a much smaller variation (13.5) and smaller mean (6.1) for the plants grown under rainfed condition with no rainout shelters (**Supplementary Figure 1**). These results suggest that there are two overlapping leaf-flipping traits governed by distinct genetic mechanisms that are manifested under two drought conditions. Soybean plants grown under rainout shelters were exposed not only to severe drought, but also to elevated temperatures due to lack of complete air-circulation under the shelter, as was possible for the rainfed crop. Thus, as expected, the two environmental conditions were not identical for expression of the trait leading to detection of two drought tolerance genetic mechanisms.

This study identified six annotated and one unannotated putative candidate drought tolerance genes; four annotated and the unannotated genes carry alternative alleles carrying nonsynonymous mutations. The rest two genes are transcriptionally regulated during drought stress. A *cis*-acting element located at the 3’-end of one of the genes is deleted, while a silencer element located 13 kb downstream from a putative candidate drought tolerance gene is tandemly duplicated in the unfavorable alleles of the two genes, respectively. Here favorable alleles refer to tolerance for drought stress, while unfavorable refers to sensitivity to drought stress. Four of the six annotated genes have been shown to be involved in drought tolerance in other plant species.

The SNP mapped to Chromosome 1 caused a nonsynonymous mutation in *GmTP1* encoding a thaumatin-like protein. The thaumatin-like proteins (TLP) are a conserved protein family implicated in abiotic stresses including drought (Faillace et al., 2021; Kumar and Kirti, 2023). *Arabidopsis* lines overexpressing a *TLP* gene from tea (*Camellia sinensis*) exhibited drought tolerance due to reduced electrolyte leakage and higher water retention capacity compared to that in wild-type plants (Muoki et al., 2021). The overexpression of *bolTLP1*, a broccoli homologue of the *C. sinensis TLP* gene, enhanced both drought and salt tolerance in *Arabidopsis* (He et al., 2021). In faba beans, *VfTLP4–3* and *VfTLP5* are significantly upregulated under drought conditions (Zhao et al., 2024). Furthermore, the transient expression of these genes in tobacco leaves generated enhanced drought tolerance (Zhao et al., 2024). The overexpression of *ObTLP1*, a *TLP* gene from basil (*Ocimum basilicum*), enhanced tolerance to dehydration induced by mannitol in *Arabidopsis* (Misra et al., 2016). In carrot, the induction of *dcTLP* was highly specific to drought stress in the embryogenic calli, seedlings and mature plants (Jung et al., 2005). These results support that *TLPs* are involved in drought tolerance and support the functional relevance of the identified *GmTP1* (*Glyma.01G165800*) gene in drought tolerance.

The leaf-flipping trait-associated SNP, mapped to Chromosome 20, caused a nonsynonymous mutation in *GmIMS1* (*Glyma20g245300*) encoding an isopropylmalate synthetase. A second SNP, ~2 kb upstream of *GmIMS1*, caused a nonsynonymous mutation in an unannotated gene. The isopropylmalate synthetase is an enzyme of the family of transferases, converting acyl groups into alkyl groups (Cole et al., 1973). This enzyme is involved in the biosynthesis of L-leucine (Yoshida et al., 2018). Mutation of *isopropylmalate synthase 1* locus in *Arabidopsis* modified the target of rapamycin (TOR) network, resulting in suppressed root hair cell development (Schaufelberger et al., 2019).

A mutation in *BZU1* results in smaller stomatal pores and higher tolerance to drought compared to wild-type *Arabidopsis* plants (Dong et al., 2018). The reduced stomatal pore size in the *bzu1* mutant resulted from the reduced accumulation of malate. The isopropylmalate synthase encoded by the nonsynonymous mutant *GmIMS1* is most likely less active resulting in reduced malate accumulation and smaller stomatal pore sizes and enhanced drought tolerance. Thus, *GmIMS1* could contribute to drought tolerance through dual functions: (i) root biology for water uptake as well as (ii) stomatal behavior for water retention.

Among the 19 genes mapped to the Chromosome 20-specific haploblock carrying the leaf-flipping trait-associated significant SNP, *GmG2MT* (*Glyma.20G245100*) encoding the glycinol 2-dimethyltransferase was found to be differentially expressed in response to drought stress (Shin et al., 2015). The expression of the gene was significantly downregulated in response to dehydration (Jahan et al., 2019; **Supplementary Table 5**), and thus, we consider *GmG2MT* as a possible candidate drought response gene.

In our GWAS using the H/T indices of 47 accessions led to the discovery of two genomic regions involved in regulation of leaf-surface moisture contents during drought stress. We identified 37 genes located in the haploblocks of these two regions on Chromosome 4 (**Figure 7**) (**Supplementary Tables 7**, **8**). Cross-referencing with the transcriptomic data (Shin et al., 2015) for differential expression during drought stress, we were able to identify two genes, *GmATB1* (*Glyma.04g088500*) and *GmPCK1* (*Glyma.04g089700*), within the haploblock located in the northern arm of Chromosome 4 (**Supplementary Table 7**). *GmATB1* and *GmPCK1* encode α-tubulin 1 and phosphoenolpyruvate carboxykinase 1 (PCK1), respectively.

In rice, expression of a *α-tubulin* gene was inhibited during water stress conditions (Sheoran et al., 2014) leading to impaired reproductive development. Downregulation of the gene was observed in a transcriptomic study in soybean (Shin et al., 2015). Similarly, overexpression of *ZmPCK2*, encoding a phosphoenolpyruvate carboxykinase in maize, showed stable yield in the mutant as compared to the wild type under drought stress conditions (Zhang et al., 2017). It was also found that the mutant type was high in relative water content with elevated transcript activity of *PCK* as compared to the wild type under simulated drought conditions induced using polyethylene glycol-6000. The upregulation of the gene was also observed in response to drought stress in soybean (Shin et al., 2015).

To understand the possible mechanisms of transcriptional regulation of *GmATB1* and *GmPCK1*, we investigated the two genes for possible differences in *cis*-acting elements. We discovered an indel of 27 base pairs at the 3’-end of the *GmATB1* gene. There are two copies of this element in the favorable allele with none in the unfavorable allele of the gene (**Supplementary Table 10**). The element contains most likely binding sites for the TFs such as DoF, ZF-HD, ARID, Sox (**Supplementary Table 10**). The differential expression of the second gene *GmPCK1* in response to drought could be associated with the downstream TTGGGCTTGGGCTTGGGCTTGGGCTTGGGC sequence carrying the TGGGCT element also known as the SITEIIATCYTC transcription factor binding site. In drought tolerant lines, four copies of the TGGGCT element were present, while among the sensitive lines, nine copies were identified (**Supplementary Table 12**).

The DNA-binding with one finger (DoF) transcription factor has been linked to its roles in stress tolerance through regulation of stress responsive genes. In potato, five cycling DoF factors (CDFs); *StCDF1/StDof19, StCDF2/StDof4, StCDF3/StDof11, StCDF4/StDof24*, and *StCDF5/StDof15*, which were also the homologs of *Arabidopsis* CDFs, were found to be engaged in variety of abiotic stresses including drought (Jin et al., 2024). Overexpressing *GmDof41* in soybean hairy roots reduced He_2_O_2_ levels and balanced proline, helping the plants tolerate drought and salt stress (Wei et al., 2023). In quinoa, zinc finger homeodomain (ZF-HD) transcription factor *CqZF-HD14* gene was found to be drought responsive (Sun et al., 2022). Transient *CqZF-HD14* overexpression enhances drought tolerance by boosting photosynthetic pigments and antioxidant defenses in quinoa. *GmZF-HD* genes were found to be differentially regulated during drought stress in soybean (Rizwan et al., 2025).

The Nuclear Factor Y (NF-Y) is a transcription factor (TF) family with high affinity and sequence specificity for the CCAAT box (Li et al., 2008). This TF is composed of three distinct subunits (NF-YA, NF-YB and NF-YC) that are strongly induced during drought stress (Li et al., 2008; Zhang et al., 2023). The NF-YA have been found to regulate drought tolerance in *Arabidopsis* (Li et al., 2008), rice (Lee et al., 2015); NF-YB in *Arabidopsis* (Sato et al., 2019), corn (Nelson et al., 2007), *Populus* (Zhao et al., 2020), and Sugarcane (Chinnaswamy et al., 2024); and NF-YC in rice (Chen et al., 2014) and seashore paspalum (Wu et al., 2018).

In Arabidopsis, the ecotype Columbia carries the SITEIIATCYTC site for binding a TCP-domain transcription factor in the cold shock domain protein gene *AtCSP4.* The SITEIIATCYTC site is absent in Arabidopsis ecotype Landsberg and is considered to contribute towards differential expression of the gene between the Columbia and Landsberg ecotypes (Nakaminami et al., 2009). These findings suggest that the indels identified in this study may have a potential role in regulating the two drought-responsive soybean genes.

We identified two putative candidate drought tolerance genes, *pGmDT1* and *pGmDT2*, from two Quantitative Trait Nucleotides (QTNs) mapped to the southern am of Chromosome 4 (**Supplementary Figures 10**-**12**). Two alleles of these two putative candidate drought tolerance genes were associated with H/T indices of a few genotypes that had >20X genome sequences. In *pGmDT1*, a nonsynonymous mutation is expected to cause structure-based functional change. Whereas a conservative in-frame three-nucleotide insertion for a serine residue at the N-terminus generated two alleles of *pGmDT2* for drought tolerance and sensitive lines, respectively (**Supplementary Figure 12**). The extra serine residue at the N-terminal region of the homeodomain protein pGmDT2 could have reduced the binding affinity of this transcription factor to promoters of the target drought responsive genes involved in drought tolerance. Homeodomain-leucine zipper protein (HAT) belongs to homeodomain leucine zipper subfamily (HD-ZIP) with its role in regulating plant growth and development and stress tolerance (Liu et al., 2022). A HAT gene from pear (*Pyrus sinkiangensis*) was overexpressed in tomato, which resulted in enhanced tolerance of tomato plant to drought and salt stress through regulation of proline metabolism and antioxidation activity, reducing ROS accumulation and maintaining cellular function, exhibiting strong potential for use in crop stress breeding (Liu et al., 2022).

GWAS relies on the extent of linkage disequilibrium (LD) in the collection of highly diverse natural accessions. Natural variants are ideal for GWAS. During evolution, LD blocks are reduced in size due to crossing over and recombination. In cross-pollinated species such as maize, LD decays rapidly because recombination occurs in each generation due to the random mating from open pollination. It is reported that the LD in maize natural variants could be around 1 to 10 kb (Yan et al., 2009). On the contrary, in the self-pollinated species, LD decays slowly because of a lack of open pollination, and the LD blocks are reported to be over 100 kb (Hyten et al., 2007). In wild species *G. soja*, LD is relatively small and less than 100 kb because some open pollination (9-19%) occurs in this species (Fujita et al., 1997). With domestication, the rates of open pollination drastically declined in the cultivated *G. max* species to 0.41% (Ray et al., 2003). Consequently, the LD among the *G. max* variants has been reported to be 90 to 574 kb (Hyten et al., 2007). Although in self-pollinated crops like soybean, GWAS identifies the genomic regions containing the genes that govern a trait, it frequently fails to identify the causal genes because the detected trait gene containing region could be >100 kb and can contain over 10 genes. In this study, the LDs were 122 to 133 kb when the number of accessions was 240. The LD values were larger, 297 to 324 kb when the sample size was reduced to 47 accessions.

GWAS enabled identification of four genomic regions containing the causal mutations for two drought traits: (i) leaf-flipping phenotype and (ii) transpiration trait monitored by a wearable sensor. Leveraging the LDs enabled us to identify 67 candidate drought tolerance genes (Myles et al., 2009; Zhu et al., 2008). We narrowed down the number of candidate genes from 67 to seven (**Table 1**), mapped to these four Quantitative Trait Nucleotides (QTNs), through consideration of the following steps: (i) if the mutation associated with a QTN can alter structure and function of a gene, (ii) if any of the genes mapped to the QTN-haploblocks are transcriptionally regulated in response to drought stress, (iii) if any of the differentially regulated genes associated with the mutations in putative *cis*-acting elements, (iv) if any of the genes mapped to the four QTN-haploblocks carry mutations that can cause alteration in the structure-functions of their encoded products, and (v) if any of the identified seven putative drought tolerance genes were previously shown to be involved in drought tolerance.

**Table 1:** The seven putative candidate drought tolerance genes identified in this study.

Sl. no.	Gene ID	Candidate gene	Mutation type	Transcriptional regulation	Trait
1	*Glyma.01G165800*	*GmTP1*	Missense	None	Leaf-flipping
2	*Glyma20g245300*	*GmIMS1*	Missense	None	Leaf-flipping
3	*Glyma.20G245100*	*GmG2MT*	Indel	Yes	Leaf-flipping
4	*Glyma.04G088500*	*GmATB1*	Indel	Yes	Transpiration
5	*Glyma.04G089700*	*GmPCK1*	Indel	Yes	Transpiration
6	*Glyma.04G174400*	*pGmDT1*	Missense	None	Transpiration
7	*Glyma.04G174467*	*pGmDT2*	Indel	None	Transpiration

In our study, the available effective SNPs for GWAS were drastically reduced when the population size was reduced from 240 to 47. The size of haploblocks containing the causal genes increased from 122–133 kb with n = 240 to 297–324 kb with n = 47 (**Figure 7**; **Supplementary Tables 4**, **5**, **7**, **8**). Application of deep (30X genome equivalent) short-read sequences allowed us to reveal indels as possible regulatory elements of two putative candidate drought tolerance genes, *pGmDT1* and *pGmDT2*. Use of the deep sequences of seven of the 47 genotypes including both drought tolerant and sensitive lines allowed us to reveal two putative candidate genes from a total of 16 genes identified from a haploblock of 324 kb QTN for the transpiration trait.

Transcriptome-wide association study (TWAS) has been shown to complement the power of GWAS for rapid isolation of candidate causal mutations of a trait. Transcriptomes can be mapped with the phenotypic variation for connecting both qualitative as well as quantitative traits with the transcript variations caused by the causal mutations (Gamazon et al., 2015; Mai et al., 2023). Such variations could be resulted from mutations in the *cis*-acting or enhancer/silencer elements of a gene. The two indels identified in this study could possibly be *cis*-elements involved in regulating the two-drought stress-responsive genes. Our approach of using the previously published transcriptomic data during drought stress facilitated identification of three putative candidate drought tolerance genes from a list of 67 genes. However, this integrative approach also represents a limitation. As the expression data were obtained under experimental conditions different from those that are under the present study, genotypic variations, environmental stress intensity and sampling stage could influence gene expression pattern, potentially leading to context specific differences. The short listed seven candidate drought tolerance gene will require validation through mutant studies. Gene knockout and overexpression studies of the identified seven candidate genes in stable transgenic soybean plants are warranted to identify the true drought tolerance genes.

The GWAS of the leaf-flipping trait was conducted using 30,823 SNPs discovered from 1X genome equivalent sequences followed by imputation of 240 accessions. As a result, the distribution of SNPs in certain regions on Chromosomes 1, 5 and 20, is less dense. This heterogeneity in marker density may reduce the sensitivity of detecting the associations of the trait variation with the possible genomics regions of these SNP sparse regions. It is essential to have deep genome coverage of short read sequences to call millions of SNPs to facilitate identification of candidate trait genes.

The GWAS of the drought traits was conducted using the Blink model implemented in GAPIT3 with three principal components (PCs) as covariates to correct for the population structure without the use of kinship matrix. Unlike the traditional mixed linear models or FarmCPU, Blink does not require an explicit kinship matrix, as it controls for relatedness through iterative inclusion of linkage-disequilibrium independent pseudo-QTNs as covariates in a fixed effect framework (Huang et al., 2018). This design effectively accounts for both population structure and kinship while avoiding the computational complexity and potential overcorrection associated with the random effect model. In some diverse soybean panels, empirical kinship heatmaps show generally low pairwise relatedness, indicating minimal recent shared ancestry within that specific sampling. However, broader population-genetic work cautions that while selfing elevates homozygosity and LD, realized kinship can still vary with breeding history and founder effects, so panel-specific estimation of kinship or conditioning on background markers remains recommended in GWAS (Kim et al., 2021; Yan et al., 2021; Priyanatha et al., 2022; Hyten et al., 2007). As a result, the degree of kinship among genotypes is relatively low compared to cross-pollinated species, reducing the need for a separate kinship matrix in a GWAS model.

The statistical power in GWAS is largely determined by sample size, allele frequency distribution, population structure. marker density and the extent of linkage disequilibrium (LD) across the genome. Due to repeated selfing over generations in self-pollinated crops like soybean, LD blocks can span over hundreds of kilobases (Aleem et al., 2024; Kim et al., 2023; Bhat et al., 2022). Even though the LD blocks can facilitate detection of large-effect loci, the mapping resolution is severely impaired when GWAS is conducted using a small population. The reduced sample sizes reduce the ability to detect minor effect alleles substantially. In our plant wearable sensor study, the sample size was reduced from 240 to 47 due to limitations in the availability of wearable plant sensors. The study of 47 accession lowered the statistical power by increasing the range of LD blocks to 297–461 kb as compared to the range from 122–133 kb, when 240 accessions were studied for the leaf-flipping trait. The SNP density was reduced from 30,823 SNPs to 17,042 SNPs, when the sample size was reduced from 240 to 47. The transcriptomic data facilitated identification of four putative candidate genes from a total of 37 genes in two SNP loci identified through GWAS of 47 genotypes. However, our approach of putative candidate genes identification based on transcriptomic data and insertion-deletion of putative *cis*-acting elements could increase the number of false negatives. When the marker density and sample size constrain mapping resolution, integrating the GWAS peaks with omics and non-omics (OnO) data can help distinguish LD-passenger false positive genes from the plausible causal target genes and guide a low-throughput validation (Kao et al., 2022. 2025).

We have integrated available transcriptomic data and deep genome sequences of a limited drought tolerant and sensitive lines to eliminate most of the false positive candidate drought tolerance genes. It’s however unknown in this process if we had eliminated any desirable drought tolerance genes as false negative. It is also worth noting that the responses of the putative transcriptionally regulated genes identified based on a previous transcriptomic study (Shin et al., 2015) were not validated by conducting reverse transcriptase-polymerase chain termination reactions (RT-PCR). Gene knockout and overexpression studies should be able to ascertain if the selected putative candidate genes contribute towards drought tolerance and we have not discarded any important drought tolerance genes as false negative.

In this study, seven putative candidate genes associated with drought tolerance were identified from the experiments conducted at a single location and single year with only two replications due to lack of abundant space under the rainout shelters. As a result, most likely environmental effects were confounded with the genetic effects leading to less effective characterization of genetic variation through the GWAS. Microclimatic differences such as variation in temperature and humidity inside versus outside the shelters, could influence phenotypic variation hindering the outcomes of GWAS. Phenotyping across multiple locations and multiple years can enhance the robustness of GWAS for identifying strong candidate genes. Unlike many other studies, we were able to identify only a few SNP loci by keeping the level of significance to lower levels as opposed to detection of many QTNs due to applications of higher levels of significance (e.g., Kaler et al., 2017; Aleem et al., 2024). Furthermore, integration of the available transcriptomic data, deep sequencing of a few genotypes, and literature search for the possible drought tolerance functions of the homologues led to identification of only seven putative candidate drought tolerance genes from four QTNs (He et al., 2021; Misra et al., 2016; Schaufelberger et al., 2019; Sheoran et al., 2014; Zhang et al., 2017).

## Conclusion

We have identified seven putative candidate genes associated with soybean drought tolerance by conducting GWAS on two key traits: (i) the leaf-flipping phenotype, a visible drought-adaptive phenotype, and (ii) the ratio of humidity to temperature (H/T) recorded by wearable plant sensors to indicate the transpiration regulation during drought stress. The leaf-flipping trait is a morphological response to drought stress, while the H/T ratio represents a physiological transpiration response during drought stress. The GWAS of H/T ratios gathered from 47 accessions revealed two candidate transcriptionally regulated drought-responsive genes encoding α-tubulin and phosphoenolpyruvate carboxykinase (PCK). The α-tubulin was shown to control stomatal opening, while PCK improves water retention by closing stomata during drought stress (Sheoran et al., 2014; Zhang et al., 2017). This application of plant wearable sensors at population level for the first time led to identification of two putative drought responsive genes that could regulate stomatal opening during drought stress. Thus, this study laid a strong foundation for dissecting the molecular basis of differential transpiration responses among the accessions during drought stress.

In this GWAS, integration of available transcriptomic data and deep short-read sequences of a collection of genotypes including both drought tolerant and sensitive lines and literature search for drought tolerance function of the homologues of putative candidate genes allowed us to uncover seven putative candidate drought tolerance genes and lay a robust foundation for developing climate-resilient soybean cultivars.

## Materials and method

### Field and plant materials

A total of 240 diverse soybean lines, comprising 172 diverse Plant Introduction (PI) lines (Valliyodan et al., 2021) collected from GRIN-Global and 68 improved soybean germplasm lines and cultivars developed at the Iowa State University were used in this study. Some of the selected PI lines were previously reported as drought tolerant lines (GRIN-Global).

Field trial was conducted at the Hinds Farm, located to the north of Ames, Iowa. The plants were grown in two environmental conditions: (i) rainfed and (ii) drought stress. The drought stress was created by mobile rainout shelters (30’ X 12’ X 48’ Gspan Rolling Prem High Tunnel, https://www.farmtek.com/cat/ft-high-tunnels-cold-frames.html) (**Figure 5A**). In each rainout shelter, 80 genotypes were randomized. All 240 genotypes were randomized and grown across three rainout shelters from one end of the field for the Replication 1and again rerandomized across the remaining three shelters for Replication 2.

In parallel, the same 240 genotypes were grown under rainfed conditions in six adjacent open-field plots, with 80 genotypes randomized per three plots replicated twice next to rainout shelters 1–3 (**Figure 5A**). In each plot of 3.5’ x 2.5’, 30 seeds of an accession were planted using a NARDI professional series precision vacuum planter that plants 4 rows at a time. The open space between the two adjacent plots was 1.5 feet. Ten rows were planted across the width of the rainout shelter with each row comprised of eight plots. The rainout shelters were also equipped with a drip-irrigation system to prevent plants from complete wilting and dying.

### Sensors

Wearable plant sensors used in this study were developed at the Microelectronics Department, Iowa State University (Yin et al., 2021; Yin and Dong, 2024; **Figure 5C**). These sensors were attached to the abaxial surface of the fully developed third leaf from the apex (**Figure 5D**). A double-sided adhesive tape was used to attach the sensors to the lower side of the leaf. The sensors were connected to the data logger using a cable (**Figure 5B**). The data loggers were connected to the power banks for electric power supply. The data loggers were equipped with 32GB SD cards (SanDisk, Fremont, CA) used for storing the temperature and humidity data collected every 30-min collected by the wearable plant sensors. For charging the power banks, it was connected to a battery that acquired electricity from a solar panel. Three sensors were attached to three individual plants of a genotype. A total of 47 lines from replication 1 (the rainout shelter # 3; Replication 1) and replication 2 (rainout shelter # 4; Replication 2) were included for this study. Some of the selected 47 lines were previously identified as drought-tolerant accessions.

### Genotyping

To identify the SNPs across all 20 chromosomes in a panel of 240 soybean lines, the whole-genome resequencing was conducted at the HudsonAlpha Institute for Biotechnology, Huntsville, Alabama. The leaf tissues were sampled using two tissue punches of 6 mm diameter per accession and stored in 96-well microtiter plates. Sequencing was conducted using the Khufu technology to obtain 1x genome equivalent DNA for each of the 240 lines. The soybean genome assembly version 4 (Wm82.v4) was used for mapping and variant calling. SNPs were imputed using STITCH (Davies et al., 2016), which is a highly accurate imputation tool specially designed for low coverage sequencing data. Additionally, it offers the advantage of not requiring external reference panel while maintaining superior imputation accuracy as evidenced by high mean imputation score (*r^2^* = 0.98).

### Whole genome sequencing of selected lines

Selected 12 genotypes identified as either extreme tolerant or extreme sensitive were sequenced to an average sequencing depth of 30X using Illumina Sequencing Platform NovaSeq6000 in the DNA facility at Iowa State University. The sequence reads were 150 base-pair paired-end sequences. An average of 30 million reads were generated for each genotype. The low-quality reads and adapter sequences were removed using Trimmomatic (Bolger et al., 2014) version 0.39 with the default parameters. The reads with less than 36 base pairs in length were discarded. Williams 82 version 4 was used as a reference genome for mapping and variant calling. Filtered reads were mapped to the reference genome using bwa mem (version 0.7.17) (Li et al., 2009) while Picard (2019) was used to remove duplicates and fix mate pairs. Finally, samtools (Li et al., 2009) with varscan (Koboldt et al., 2009) were used for variant calling using the default parameters.

### Association study

A genome-wide association study was conducted using the imputed SNP dataset from 240. The initial 71,560 SNPs after imputation were filtered for heterozygosity (> 5%) and minor allele frequency (< 5%) resulting in a total of 30,823 high quality SNPs for conducting GWAS using the phenotypic scores of the leaf-flipping trait and 17,042 SNPs identified from analyses of 47 genotypes for GWAS with the H/T indices. Genome Association and Prediction Integrated Tool (GAPIT) (Lipka et al., 2012) in R Studio version 4.3.1 was used for GWAS. BLINK model was used in GAPIT, along with three principal components set as covariates for GWAS (Huang et al., 2018). To control for multiple testing, we applied modified Bonferroni-corrected significance threshold of p < 1 x 10^-6^. This threshold was selected instead of a permutation-derived cutoff because permutation analysis (10,000 iterations, shuffling genotypes) yielded a highly comparable empirical threshold (1.28 x 10^-6^) (**Supplementary Figure 13**). The close agreement between the two approaches supports the use of Bonferroni-adjusted threshold while maintaining computational efficiency.

### Annotation of the putative candidate gene

The haploblocks harboring the significant SNPs were investigated for putative candidate genes associated with leaf flipping or transpiration rate traits. For identification of the putative candidate genes, the gene coordinate file of soybean genome assembly version 4 (Wm82.v4) was used. We explored if the significant SNPs were located within gene coding sequences. If SNPs were located within coding regions, then the SNPs were further examined for the type of mutations and if they can alter the structure-function using the bioinformatic tools from the National Center for Biotechnology Information (NCBI). The putative candidate genes of the haploblocks were further studied for their possible expression in response to drought stress using the previously reported transcriptomic data (Shin et al., 2015).

### Analysis of cis-regulatory elements

The differentially expressed genes with no mutations in the coding sequence were investigated for possible loss or gain of *cis*-acting elements. To identify such possible elements, we used the 30x genome equivalent sequences of the 12 selected genotypes that were either drought tolerant or sensitive. The indel sequences were used to locate any regulatory element for binding transcription factors using the webtool PlantPAN 4.0 (https://plantpan.itps.ncku.edu.tw/plantpan4/index.html) with the promoter analysis option. The sequences were uploaded as a single line in the FASTA format, one sequence at a time, and only default parameters were used along with *Arabidopsis thaliana* and *Glycine max* as species. The resulting output file was used for downstream analysis and interpretation.

### Annotation of the SNPs

The variant call file from the 12 genotypes was used for annotation of the SNPs. SnpEFF version 5.2 was used for annotation using the Williams82 version 4 genome assembly, coding sequence and protein sequence file in fasta format and gene annotation file in gff3 format (Cingolani et al., 2012). The SnpEFF was used for building the database using all the files mentioned and, consequently, the variant file was annotated. After annotation, the mutations that have high impact like missense mutation, stop gained, conservative in frame insertion/deletion, disruptive in frame insertion/deletion, frameshift variant were selected for impact on the function of the gene.

## Data Availability

Data supporting this article are available in the **Supplementary Table** along with this manuscript as well as deposited in the public data sharing platform Zenodo (https://doi.org/10.5281/zenodo.17535405). The detailed scripts used for processing reads and variant calling can be found at https://github.com/atitparajuli2020/soybean_drought_project.git.
