# Supplementary material for: Deep short-read sequences facilitated identification of seven putative drought tolerance genes in a genome-wide association study in soybean
Source: Front Plant Sci. 2025 Dec 16;16:1661547. doi: 10.3389/fpls.2025.1661547 (PMC12750341; doi:10.3389/fpls.2025.1661547)
Supplement: Supplementary file 1 [file SupplementaryFile1.docx]

**Supplementary Figures 1 – 14**

**
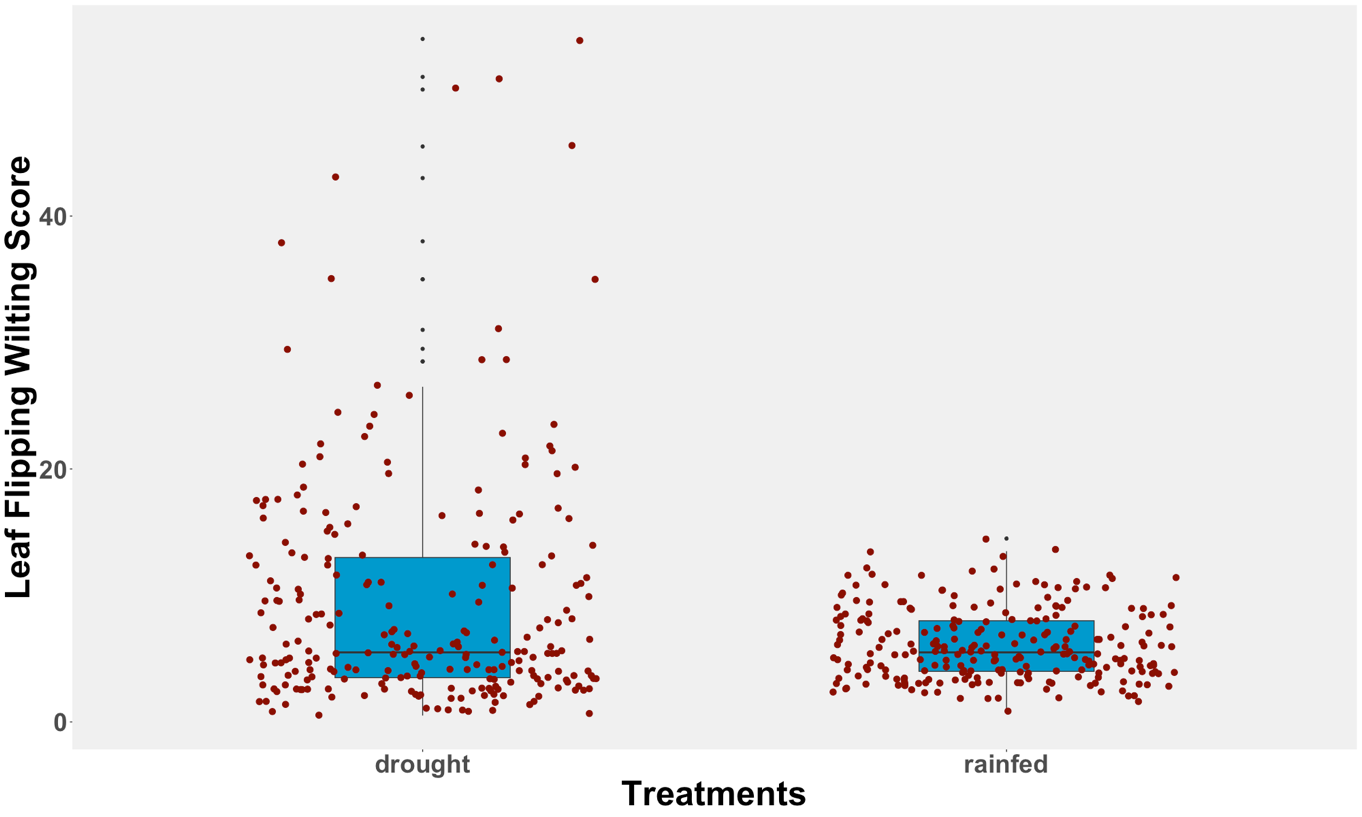
Supplementary Figure 1**: Distribution of leaf-flipping scores among the 240 soybean accessions grown inside the rainout shelter (drought) and as rainfed control.


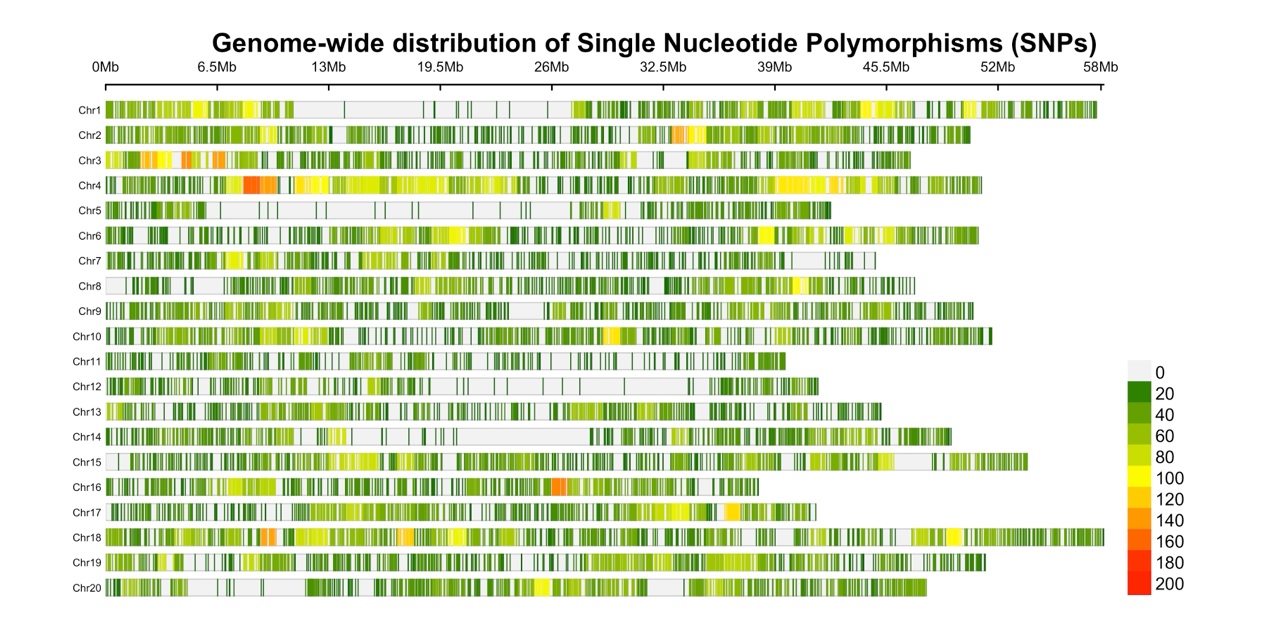


**Supplementary Figure 2**: Genome-wide distribution of 30,843 single nucleotide polymorphisms (SNPs) among the 240 genotypes. The SNPs are uniformly distributed across all but Chromosomes 1, 5, 8, 12, 14 and 20.

**Supplementary Figure 3**: Phenotypic variations under the rainout shelters of each of the three genotypic classes for the SNP alleles mapped to Chromosome 1. The SNP causes nonsynonymous mutation in *GmTP1* (*Glyma.01G165800)* gene encoding a thaumatin like protein (**Figure 4**). The X-axis represents the three genotypic classes from two alleles of the SNP. The frequency of the reference (Williams 82) homozygous (GG) class is 0.28, and that for the alternate homozygous (TT) class is 0.72. The leaf-flipping phenotypes of the genotypes are presented on Y-axis.

**Supplementary Figure 4**: Phenotypic variations within each of the three genotypic classes for the SNP alleles mapped to Chromosome 20. The SNP causes nonsynonymous mutation in *GmIMS1* (*Glyma20g245300*) gene encoding an isopropylmalate synthase (**Figure 4**). The X-axis represents the three genotypic classes from two alleles of the SNP. The frequency of the reference (Williams 82) homozygous (CC) class is 0.85, and that for the alternate homozygous (TT) class is 0.15. The leaf-flipping phenotypes of the genotypes are presented on Y-axis.

**Supplementary Figure 5**: Phenotypic variations of leaf-flipping phenotype under no rainout shelters within each of the three genotypic classes for the SNP alleles mapped to unannotated *GmHk_20G059303* gene on Chromosome 20. The X-axis represents the three genotypic classes from two alleles of the SNP. The frequency of the reference (Williams 82) homozygous (TT) class is 0.6, and that for the alternate homozygous (CC) class is 0.4. The phenotypic variation of the genotypes is presented on Y-axis.


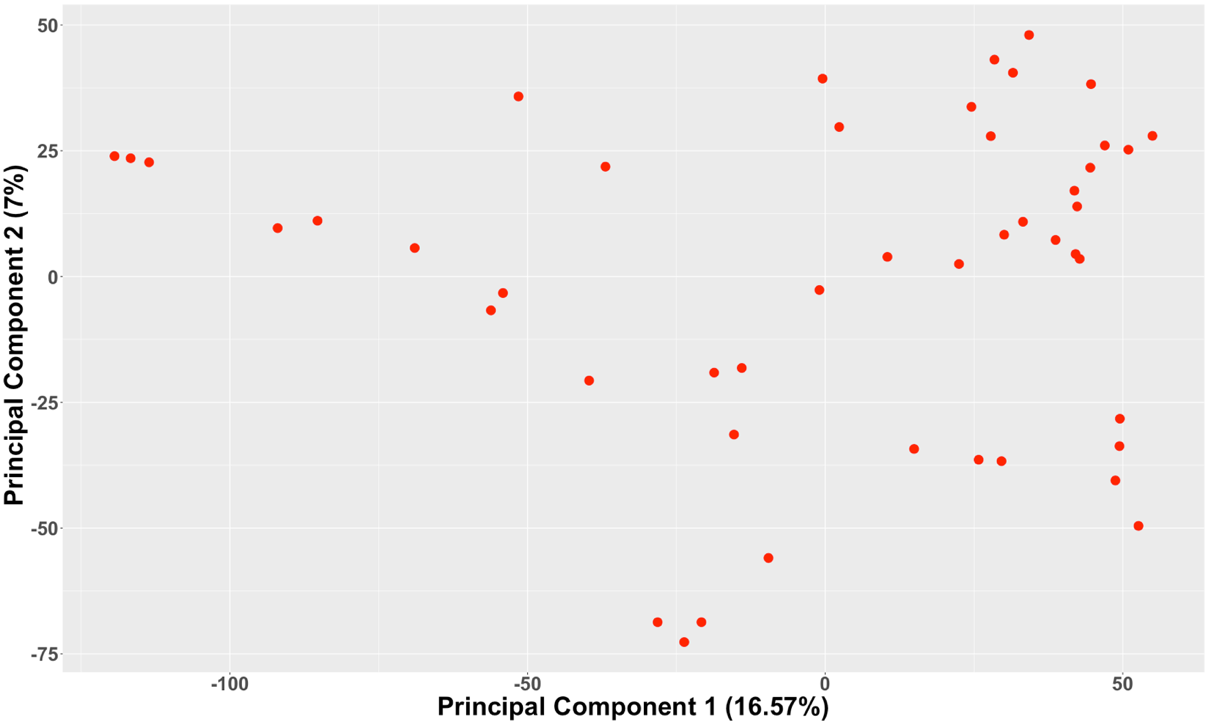


**Supplementary Figure 6:** The population structure revealed by the scatterplot of the 47 genotypes using the first two principal components that explain 23.57% of the genotypic variation based on 17,042 SNPs.

**Supplementary Figure 7**: Phenotypic variations of each of the genotypic classes for the SNP alleles mapped to the northern arm of Chromosome 4. The X-axis represents the genotypic classes from two alleles of the SNP mapped to Chromosome 4. The frequency of the reference (Williams 82) homozygous (AA) class is 0.13, and that for the alternate homozygous (GG) class is 0.87. The phenotypic variation as H/T ratios of the genotypes are presented on Y-axis.

>Gm04:7583650-7586692_alpha tubulin

GGGTTAACCCAACCCTAGTAAATTTCCAACCCCACATTTCAAATTTAGCCACCCTCATTTCTGCGCATATATAACCCCCAACA **A**CAATGGTGACAGACTCACAATCGAACGGGAAAAGGCGTCTTCATAAACGCCGCCCAAAACTTCTCATACCCTTTTATTCATTCCATTCGTTTCTAGGGTTTTCTTTTCTTTGCCGAGAAA**ATG**AGAGAGTGCATTTCGATCCACATTGGCCAGGCCGGTATCCAGGTTGGAAACGCCTGCTGGGAACTCTATTGCCTTGAACACGGCATTCAGGTATGGTTTCAAATGATTTTATTAGGTTTCAGTTTGGGCTTCGGCCTGGGCTTGGGCTTGGGTTTTAATGTTCTTTTTTTTTTTTTTGTGTTCAGCCCGATGGACAGATGCCGAGTGACAAGACCGTTGGCGGAGGAGACGACGCCTTCAACACATTTTTCAGCGAGACCGGCGCCGGAAAGCACGTGCCCCGCGCCGTCTTCGTGGATCTTGAACCCACCGTGATCGACGAAGTCCGAACCGGTGCGTACCGGCAGCTCTTCCACCCGGAGCAGCTGATCAGCGGCAAGGAAGACGCCGCCAACAACTTCGCGCGTGGCCACTACACCATCGGGAAGGAGATCGTCGATCTCTGCCTCGACAGGATCCGAAAGCTCGCCGACAACTGCACCGGTCTCCAGGGTTTCCTCGTCTTCAACGCCGTCGGTGGCGGCACCGGTTCTGGCTTGGGCTCGCTTCTCTTGGAGCGTCTCTCCGTTGACTATGGAAAAAAGTCAAAGCTCGGTTTCACCGTTTATCCATCCCCTCAGGTTTCGACCTCGGTCGTGGAACCCTACAACAGCGTCCTTTCAACGCATTCTCTCCTCGAACACACCGATGTTGCTGTGCTTCTCGACAACGAGGCAATCTACGACATTTGCAGGAGATCTCTTGACATCGAACGCCCTACCTACACCAACCTGAACCGTCTCGTTTCCCAGGTATACAGTCAAACCTTTTTCATAACCGAATTTTCAGTCGTTAATTTTAACCGTTACTTAAACATAGAAATGGTTAGGTTTAAATGCGTGATTGAAATCTGATTTTAATAAGATTTTAATGTGAAGATTTTGGGTTACACACTTTATGAATGTGTGTGTATTCGACTTAAAAATTCAAATTCGAATGAATCTGGATTTCATGTAATGTATTTCTCCATGCATTCCCATAGTTTGTAATTCGGGTGGCTTACTACTAATATCGATAAATCTAAATAAAACACATTAAAATCTGATTGTTACGATGAACTGAATCTTAATCTGTTTGGAATTGGAATTTCATTTGGTGTTTGATTTTGTCTAATTTAAAATTTAAAATTTTTGTAGGTGATATCATCCCTCACTGCCTCTTTGAGGTTTGATGGAGCACTGAATGTTGATGTGACTGAGTTTCAAACCAACTTGGTTCCTTACCCAAGGATCCATTTCATGCTTTCCTCGTATGCCCCTGTTATCTCTGCCGAGAAAGCATACCATGAGCAGCTTTCCGTTGCTGAGATCACCAACAGTGCCTTCGAGCCATCCTCCATGATGGCAAAGTGTGACCCTCGCCACGGCAAGTACATGGCGTGTTGTTTGATGTACCGGGGCGATGTCGTGCCCAAGGATGTGAATGCTGCTGTGGCCACCATCAAGACCAAGAGAACCATCCAGTTTGTGGATTGGTGCCCCACTGGGTTCAAGTGTGGTATAAACTATCAGCCTCCTACTGTTGTTCCTGGAGGTGACCTTGCCAAGGTGCAGAGGGCTGTGTGCATGATTTCGAACTCCACTAGTGTGGCTGAAGTGTTCTCCAGGATTGATCATAAGTTTGACCTCATGTATGCGAAGAGGGCCTTCGTGCACTGGTATGTGGGTGAAGGTATGGAAGAGGGTGAGTTCTCAGAGGCTCGTGAGGACCTTGCTGCTCTTGAGAAGGATTATGAGGAGGTTGGTGCCGAGTCTGGTGATGGAGATGATGATGGTGAAGGAGATGATGATTAT**TAG**AGTGTCTGAAATATCGTGTGTTTGATGTCTACTTTTTCGTCTTCTGTCGGACATGTGTGGTTTTTGAATGATGATTGTTATCAAATATATGTGCTATGTATAGCACAGAAGTTTCTATATATCTGTTCCCTTCTATTACCAAT**A**TTTTCGGTCTTGTCTCTTCGGTGTTTTGGTGATGCATCGTTTAATTAATTGTTGCTAATGTTTGAGAAAATGGGGGTCTGCTCGTGCCATCTTTGTGAAGAATTAGTGGCAAAACTCATTTTATTTGGGTTCTCAATTCTGAAGACAGACGGTAGAAAGAAATGCCATTTGGAGGGTCTTCTATCGTTATGTTTAATTTATAATGTTGTAAACTAAACTTCGAACATGAATTATCAAAAGGAACACAAAACTATACTACATTGAAACTAAAAATTACTTTTCACCAATTCAACATCTGGACTTTCCAACAAATAGTTAAAACCACTTAGGCTTTTTTAAACAAATGTAGGTTTATATATTTTTACAGAGATCCATGTGTGTATTATTATTTAACCAATTATAGGTTTAATATCAAATTAGATCCATGTTCGTTAAAATTTATTATTTTTATTTTGTAGGACATATGTTAATATATTTTTTTAACCTGTTTATTTTATAATTTTCTTGTTGGCTTGTCATTTAATGTAGATGTAATTGTATCAACAACAGTTTAATTCTGATTGGACTACTGAACCTGTGAATGAACTTTATTAATTTAATAA**CCGGTATGAACTTTATTAATTTAATAACCGGTATGAACTTTATTAATTTAATAA**TTGCCTGAAACGTGTGTCTAAAGAAATCTTAAAATATACAAAAATACACTATCCCTTCCTTTTACTTGGCCTTTCATATATGCGCCCAGCCTATAGCTAAATAAAACCAGATATAATAAAATAATAAAATAGTAAAAGTTATACACAATAAATATATACTATTTTATCTTTTCCTTTGTTTTTCCCTATTCAGGGGACAGA

**Supplementary Figure 8**: The sequence of *GmATB1* encoding an α-tubulin showing transcription initiation site at 84 base pair (**A**CAATGGT), open reading frame at 205 base pair (**ATG**AGAGAGTGC), stop codon at 2,040 base pair (GATGATGATTAT**TAG**), transcription stop site at 2,186 base pair (ATTACCAATA) with the insertion sequence at 2,789 base pair (CCGGTATGAACTTTATTAATTTAATAA, 2 copies). The favorable allele (tolerant genotype) which is similar to the reference genome contains the two copies of the insertion sequence while the unfavorable allele (sensitive genotype) contains no copy of the insertion sequence. The above sequence is taken from the favorable allele that shows presence of two copies of the insertion sequence.


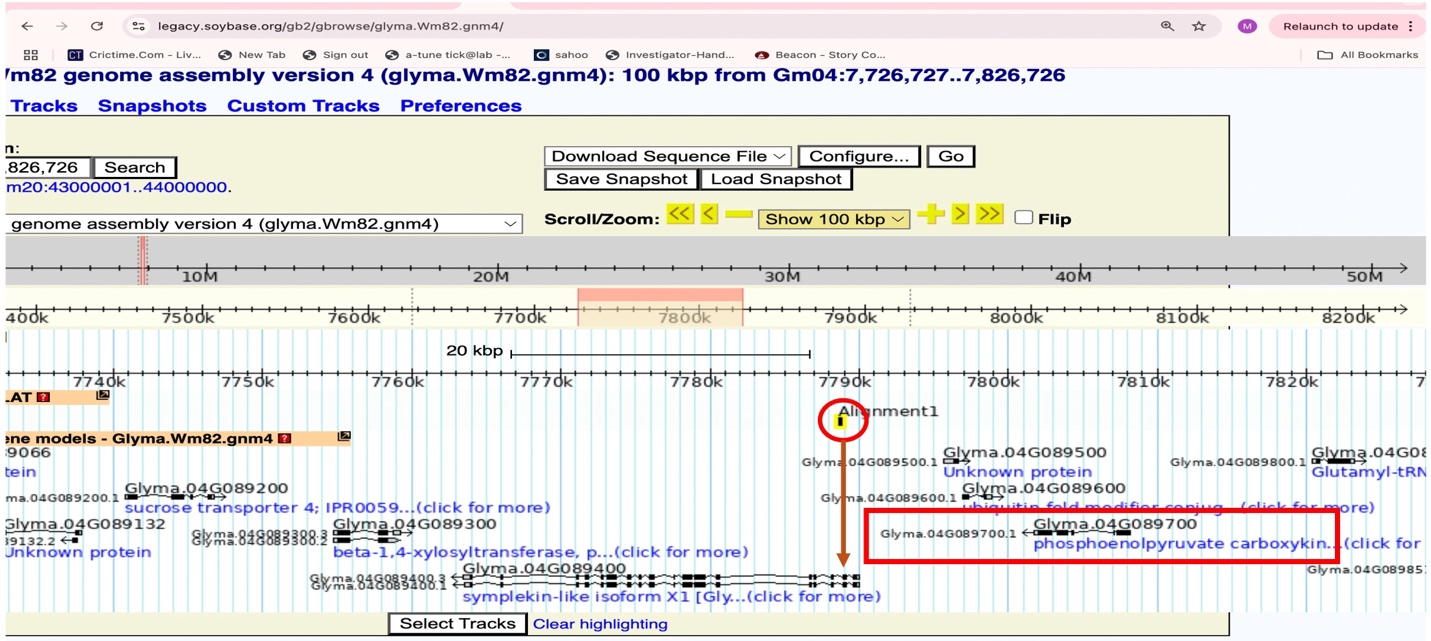


**Supplementary Figure 9**: The differentially expressed gene, ***Glyma.04G089700,*** termed *GmPCK1* encoding a phosphoenolpyruvate carboxykinase (PCK) (in redbox below) is most likely regulated by a silencer element (**ttgggc**ttgggc**ttgggc**ttgggc**ttgggc**) located at the 3’-end (shown with a red circle). The element is in an intron of a downstream gene (shown by a maroon arrow). The element has a repeat of six nucleotide base pair as ‘tgggct’ with nine copies in drought sensitive lines and five copies in the drought tolerant lines. This is a screenshot of the GBROWSE located in SOYBASE (https://www.soybase.org/tools/browsers/).

**A)**

ATATTTAGTCTTATTATTATCACAGAAAATGTCCTTTGTGTCTCTACCTCTGCATCGTCCCACGTGGCAGCTGAGTTGAG

ATATCAAGCAGAAGAAGCACTCTGGTTCTGAGGCGCAACCTCGTCCCAACAACC**ATG**GCGGTCCCTGCTACATCCTCAGC

CCCTGTCCTTCGACCCATCC(**G/C)**CACCAATCACTCCTTCTCTTCCCCTTTCCGCTCTCTCCCCTCTCCCGCCGCCAGAAAAC

CCCTAACCGTCTTCGCCATGGCCCCCAAAAAGAAGGTACAAATTCCTTATTTAATCCCTCACTAATCTTTTATTTCGTAA

ATGTCCAGTTCCACAAATTTCACGTTTGAGATGCCTAATTTGTGGCGTGAAAAGATTGTGTTAAAAAATCTCAAATTTAG

CAGTGGTATGTTCAAAATTGTGTGTATGAAAAATGTTAGAAACACAGATGCTTTTTTTTTTATTGGTTCAAATCTATTGA

AAATTATAAAATTTTATAGGTCTCATTTTATTTAATGATTCCTTCTCGTGATTGTTTGGTTTTTAATAAGTTTTAACCAA

TAAAAAAAAAAATAAACATGTTTTACTAATCGGAAAAATGCTAGTGATGAGCAATGGCGGATACAGAATCCTAAGTTAGT

GGGAATAAAAAAGCTTACTTGCCACTAGTGTTACTGGAGAGTGAGAAATTAGAGAATACTGAATTCAAATATACTAAACT

AGAGGGAGTAAAACACCAGAATTTAAGACATTTAGATGTAACTTTTCAAATTCAAGGTGCCCATAATTTCACACTGAATT

ATATATATAGGTCTGTGAATGGTAATTATTTTTGTTAACGAGAAAGATTAGAATCCACAATCTTTTCTTCTTTCTCCCTT

CTTCTTCTATCAGCAACCTTGATGAATTAAGTGGAAACTCAAAGGTTAAGATTTGATTTTAGTTATGGAATTGAACTTGG

TAGGTGAACAAGTACAATGATAAGTGGAAGAAGGAGTGGTTTGGAGCGGGGATATTCTACGAGGGAAGCGAGGAGGTGGA

GGTGGACGTGTTCAAGAAGATAGAGAAGAGGAAGGTTTTGAGCAACGTGGAGAAAGCTGGCTTGCTCTCCAAGGCAGAGG

ACTTGGGATTCACGCTCTCTTCCATAGAGAAGCTCGGCGTCTTCTCCAAAGCCGAGGAGCTCGGCTTGCTCAGCTTGCTC

GACCGAGCCGCCAGCTTCTCCCCCTCCCTCCTCGCCTCCGCCGCCCTCCCCGCCTTCGTCGCTGCCATTGCCGCCATTGT

GCTCATTCCCGACGACTCTGCCACCCTCGTCGCCGTCCAGGCTGTTGTTGCTGCCGCGCTCGGCGTCGGAGCCGTCGGGC

TTTTCGTTGGCTCCGTCGTGCTCGGCGGCTTGCAAGAGGCTGAT**TGA**TTGAATGGTGTGGGAATGGTATGTGGATGAATT

CATCTTAGATTTTTTTTTTTTGTCTTTGTAGTTGTAGAATTGAGGAGGAGAGATTATGGCAGTTTCTTTTGGTGTAAAAA

AATTGTAATAAAAAAAATGAAATGACAATAATTGACTGGAAATGGTTGCATACAACAGAATGTAGTAGTGTCTCTGTCTC

TTGTGAATGAATTGCTGTTTTC.

**B)**

Desirable Allele: MAVPATSSAPVLRPI**R**T

MAVPATSSAPVLRPI T

Undesirable Allele: MAVPATSSAPVLRPI**P**T

**Supplementary Figure 10**: A) The nucleotide sequence of the uncharacterized protein gene *Glyma.04G174400* identified from the southern haploblock region of the Chromosome 4 carrying a nonsynonymous mutation (G/C). B) The G to C mutation in A) resulted in an amino acid substitution from arginine (R) to proline (P).

Top of Form

| *Glyma.04G174400* 79 KIEKRKVLSNVEKAGLLSKAEDLGFTLSSIEKLGVFSKAEELGLLSLLDRAAS.[2].PSLLASAALPAFVAAIAAIVLI 155  [XP_006842101](https://www.ncbi.nlm.nih.gov/protein/586687061?report=GenPept) 82 KLEKKKVLSNVEKLGFLSKAEELGFTLSSIEKLGFLSKAEELGLLSLVEQAAG.[2].PSALAASALPLSVAAVAAVVVI 158  [XP_002271953](https://www.ncbi.nlm.nih.gov/protein/225456666?report=GenPept) 78 NLEKKKVLSNVEKSGLLSKAEELGFTLSSIEKLGVFSKAEDLGLLSLLEKAAS.[2].PSALASAALPIFVAAIAAIVLI 154  [EFH63815](https://www.ncbi.nlm.nih.gov/protein/297333397?report=GenPept) 83 KLEKRKVLSNVEKSGLLSKAEELGLTLSSLEKLKVFSKAEDLGLLSLLENLAG.[2].PAVLASAALPALTAAIVAVVLI 159  [Q0JLN9](https://www.ncbi.nlm.nih.gov/protein/122228669?report=GenPept) 72 KLERRKVLSTVEKAGLLSRAEELGVTLSSLEELGLLSKAEDLGLLSLVEAAAA.[2].PDALASVSLPLLVAAIAAVVLV 148 [Japanese rice](https://www.ncbi.nlm.nih.gov/Taxonomy/Browser/wwwtax.cgi?id=39947)  [CDM83289](https://www.ncbi.nlm.nih.gov/protein/669029173?report=GenPept) 75 KLEKRKVLSSVEKAGLLSKAEELGVTLSSLEKLGLLSKAEDLGLLSLVESAAT.[2].PAVLASLSLPLLVASIATVVFV 151  [XP_005847850](https://www.ncbi.nlm.nih.gov/protein/552830120?report=GenPept) 121 RIQQLKLLSKLEQSGLLSLAEKNGVTLSKLEQSGLLSAAESLGVVSLLGDRNF PGTLYALATALLVAGPASVYFL 195  [Q9SD79](https://www.ncbi.nlm.nih.gov/protein/75203190?report=GenPept) 45 RVEQLKLLTKAEKAGLLSLAEKSGFSLSTIERLGLLTKAEEFGVLSAATNPET PGTLFTLSLGLLLLGPVFAYVV 119 [thale cress](https://www.ncbi.nlm.nih.gov/Taxonomy/Browser/wwwtax.cgi?id=3702)  [XP_009416833](https://www.ncbi.nlm.nih.gov/protein/695057149?report=GenPept) 71 NVEKLRLLTKAEKAGLLTAAENFGLSLSTVERLGLLSKAEELGVLSAATDPAT PGTLLSISLALLVLGPVCVFVV 145 |
| --- |
| *Glyma.04G174400* 156 PDDSATLVAVQAVVAAALGVGAVGLFVGSVVLGGLQE.[1]. 193  [XP_006842101](https://www.ncbi.nlm.nih.gov/protein/586687061?report=GenPept) 159 PDDSIALVAAQAVLAGALGVAAAGAFLGSVVLGGLQE.[1]. 196  [XP_002271953](https://www.ncbi.nlm.nih.gov/protein/225456666?report=GenPept) 155 PDDSAALIAVQALIAGALVVGATGLVVGSVVLGGLQE.[1]. 192  [EFH63815](https://www.ncbi.nlm.nih.gov/protein/297333397?report=GenPept) 160 PDDSTTLVVAQAVLAGALALTGVVLLVGSVVLDGLQE.[1]. 197  [Q0JLN9](https://www.ncbi.nlm.nih.gov/protein/122228669?report=GenPept) 149 PDDSAALVALQAVLAAVLLAAAAGLFVGSVVLAGLQE.[1]. 186 [Japanese rice](https://www.ncbi.nlm.nih.gov/Taxonomy/Browser/wwwtax.cgi?id=39947)  [CDM83289](https://www.ncbi.nlm.nih.gov/protein/669029173?report=GenPept) 152 PDDSTLLVTVQTVVATLFAAVAAGLFVGSVVLDGLQD.[1]. 189  [XP_005847850](https://www.ncbi.nlm.nih.gov/protein/552830120?report=GenPept) 196 PDDSTALVAVQAVIALSCIAGGSAAWGGATLLSSLQK.[1]. 233  [Q9SD79](https://www.ncbi.nlm.nih.gov/protein/75203190?report=GenPept) 120 PEDYTWEVVIQVLVALLSVLGGSAAFAASGFVSNLQK.[1]. 157 [thale cress](https://www.ncbi.nlm.nih.gov/Taxonomy/Browser/wwwtax.cgi?id=3702)  [XP_009416833](https://www.ncbi.nlm.nih.gov/protein/695057149?report=GenPept) 146 PEQYPWEVALQIIVALVCVVGGSAAFAASNFVSNLQK.[1]. 183 |

Bottom of Form

**Supplementary Figure 11**: The uncharacterized protein encoded by the gene *Glyma.04G174400* contains a conserved DUF1118 domain.

Wang J, Chitsaz F, Derbyshire MK, Gonzales NR, Gwadz M, Lu S, Marchler GH, Song JS, Thanki N, Yamashita RA, Yang M, Zhang D, Zheng C, Lanczycki CJ, Marchler-Bauer A. The conserved domain database in 2023. Nucleic Acids Res. 2023 Jan 6;51(D1):D384-D388. doi: 10.1093/nar/gkac1096. PMID: 36477806; PMCID: PMC9825596.

**A)**

TTGACCCGTATTATAAACCCAGGATTCCCTCTTCTTCCATGAACACCCCCCCTCAACAAAAGGAAACTCATTCACCTTCT

TCTCTCTCTCTCTCTCTCTCTCTCTCTCTCTCTCTCTCTCTCTCTCTCTCTCTCTCTCTCTCTCTCTCTCTCTCTCTCTC

GGTCTCATACACCACCTTCCATTTTCCTCCTCCAACTCCAACCCCTGCTTCTCTTATTTATATATATATTTCTCATTCCC

CGTCTTCTGAGAGTGATGGGTGTTTGCTACCGTTGTTCCCACACCTCGCACATCACCGCCATCATCATCACCAGCTTCTT

TCGCCACAAGCCTTAGAATTATACTATTATCATTCACTCACACCCTCCTCCTCCTCCCCCTTATTTCAACAACAACCGTT

GACCACATCACAATCACAATCACATTGTCTTCCTCTGAGGATCCAACCTTCTTCACATGGCGGGTGGGAGCGTCTTCTGC

TCC(**A/ACTT)CTT**CTT**CTT**CTTCTAATTTAACAACAACCACTCAGCCTCCTCTCGATTCTCTTTTCCTCTCTGCCTCTT

CCCCTTTCCTCGGTACCTTTCCTTCTTCTGCTGTTTTATTTTTTTCTTGTTTAGTTTATAAAGTCTCGTGTCAAGCTAAG

TTGGTCGTTTAGAGTGAGTTACTGTTAATTATTTTAAATTGTCTGTTTCTATATTCGATTCTTACAAATAAAAAAACTAC

TTAATTTAAGTGATCGAAATCAACTAATTCAGATTCACAACATCAGTTAAAAACTGAAAAGTACATCTTACACTTACCCC

CCAGTTCTGATTGACCCAAATATAATTTACTAACATGGGTGGATAGAATAGGACTTTGGAGTAGTCTAAATTATTAATCT

TTTGGTTGTTTGTTGTTATTTTTTCAGGTTCAAGATCTATAGTGAGTTTTGGAGATGTTCAAGGAGGGAAGGGGTGCAAT

GACTCGTTCTTCAGTTCCTACGATGAGCATGGGGATCATGAGGACATGGACGAGTACTTCCACCAGCCAGAGAAGAAGCG

CAGGCTCTCGGTAGAGCAAGTGCGGTTTCTTGAGAAGAGCTTTGATGAGGAGAACAAGCTCGAACCTGAGAGGAAGATTC

GGCTAGCCAAGGAACTCGGCTTGCAGCCGAGGCAGGTGGCGATTTGGTTTCAGAACCGCCGCGCGCGGTGGAAGACCAAG

CACCCTGCAGGCTAG

**B)**

Desirable Allele MAGGSVFCSTSS-SSNLTTTTQPPLDSLFLSASSPFLGTFPSSAVLFFSCLVYKVSCQAK 59

Undesirable Allele MAGGSVFCSTSSSSSNLTTTTQPPLDSLFLSASSPFLGTFPSSAVLFFSCLVYKVSCQAK 60

************ ***********************************************

**Supplementary Figure 12**: The sequence of a homeobox-leucine zipper HAT5-like protein gene (*Glyma.04G174467*) from the haploblock of the southern arm of Chromosome 4 with a CTT insertion (A/ACTT) for a serine residue in the drought sensitive undesirable allele (**Supplementary Table 8**) (A). B) Insertion of a serine residue at position 13 in the undesirable allele (drought sensitive) is shown.


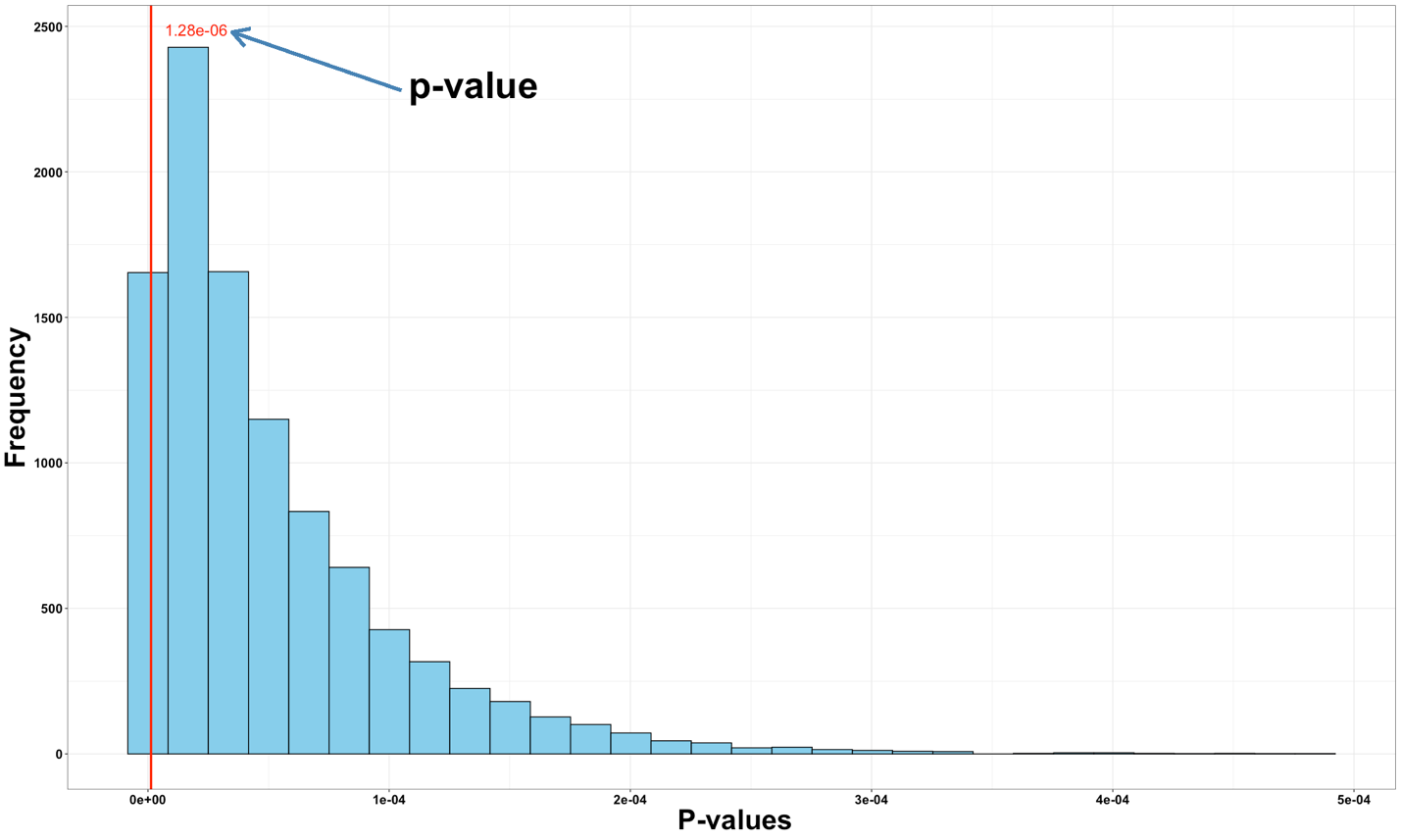


**Supplementary Figure 13**: Distribution of *p*-values after 10,000 iterations of the permutation test. The p-value at 0.05 quantile after the 10,000 iterations is indicated by the arrow. This *p*-value threshold is highly comparable with the selected Bonferroni-adjusted p-value of 1x10^-6^.
